# Phytochemical Analysis and Antiproliferative Activity of *Ulex gallii* Planch. (Fabaceae), a Medicinal Plant from Galicia (Spain)

**DOI:** 10.3390/molecules28010351

**Published:** 2023-01-01

**Authors:** Lucía Bada, Renato B. Pereira, David M. Pereira, Marta Lores, María Celeiro, Elías Quezada, Eugenio Uriarte, José Gil-Longo, Dolores Viña

**Affiliations:** 1 Group of Pharmacology of Chronic Diseases (CD Pharma), Molecular Medicine and Chronic Diseases Research Centre (CIMUS), Universidade de Santiago de Compostela, 15782 Santiago de Compostela, Spain; 2 Department of Pharmacology, Pharmacy and Pharmaceutical Technology, Faculty of Pharmacy, Universidade de Santiago de Compostela, 15782 Santiago de Compostela, Spain; 3 REQUIMTE/LAQV, Laboratório de Farmacognosia, Departamento de Química, Faculdade de Farmácia, Universidade do Porto, 4050-313 Porto, Portugal; 4 Laboratory of Research and Development of Analytical Solutions (LIDSA), Department of Analytical Chemistry, Nutrition and Food Science, Faculty of Chemistry, Universidade de Santiago de Compostela, 15782 Santiago de Compostela, Spain; 5 CRETUS Institute, Department of Analytical Chemistry, Nutrition and Food Science, Campus Vida, Universidade de Santiago de Compostela, 15782 Santiago de Compostela, Spain; 6 Department of Organic Chemistry, Faculty of Pharmacy, Universidade de Santiago de Compostela, 15782 Santiago de Compostela, Spain; 7 Instituto de Ciencias Químicas Aplicadas, Universidad Autónoma de Chile, Santiago 7500912, Chile

**Keywords:** *Ulex gallii*, antitumoral, ultra-high-performance liquid-chromatography, metabolomics tools, flavonoids

## Abstract

The genus *Ulex* comprises thirteen accepted species of perennial shrubs in the family Fabaceae. In Galicia (Spain) many of these are considered spontaneous colonizing species, which are easy to establish and maintain. Among them, *Ulex gallii* Planch. is used in traditional medicine for the same anti-infective, hypotensive and diuretic purposes as *Ulex europaeus* L., which is the most studied species. Likewise, some studies have described the antitumoral properties of several species. However, there are few scientific studies that justify the use of *Ulex gallii* Planch. and nothing has been reported about its composition to date. In our study, the entire plant was extracted with methanol and the crude extract was subjected to liquid phase extraction with distinct solvents, yielding three fractions: hexane (H), dichloromethane (D) and methanol (M), which were subsequently fractionated. The dichloromethane (D5, D7 and D8) and methanol (M4) sub-fractions showed antiproliferative activity on A549 (lung cancer) and AGS (stomach cancer) cell lines, and caspase 3/7 activity assessment and DNA quantification were also performed. Targeted analysis via UHPLC-QToF, in combination with untargeted analysis via MS-Dial, MS-Finder and Global Natural Products Social Molecular Networking (GNPS), allowed us to tentatively identify different metabolites in these sub-fractions, mostly flavonoids, that might be involved in their antiproliferative activity.

## 1. Introduction

**Figure 1:**
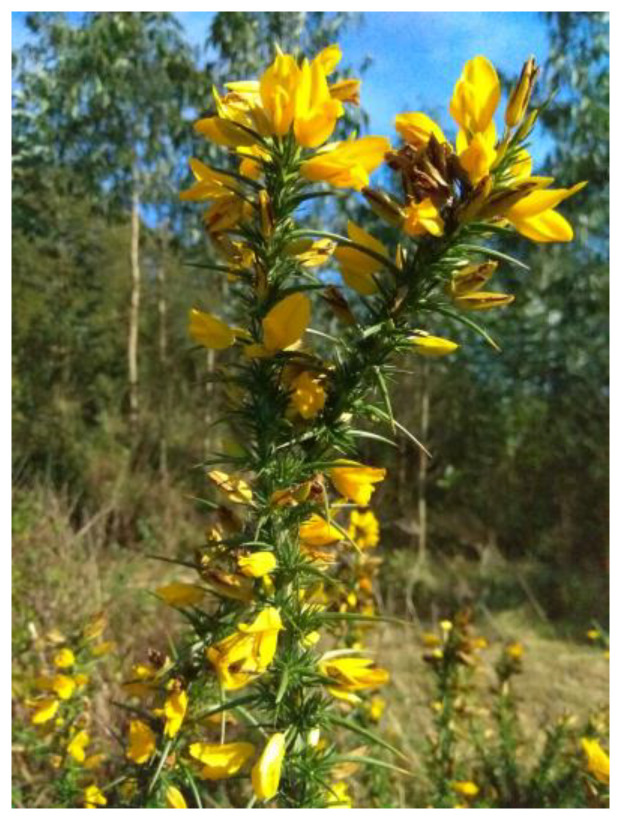
Image of the sample collected and identified as *Ulex gallii* Planch.

The genus *Ulex* contains thirteen shrubby and spiny species in the family Fabaceae, which are commonly known as gorse. The genus is native to Western Europe and Africa and most species can be found on the Iberian Peninsula. In other regions (North America, South America, New Zealand and Australia) it has been introduced as an ornamental plant. In Galicia (Spain), many of these species are considered spontaneous colonizing species, being non-invasive and easy to establish and maintain. Due to its wide distribution and large quantity, gorse has been used for many years as a “mattress” for livestock, as compost to fertilize fields and sometimes it has been treated like waste [1]. Currently, flowers are used for the preparation of desserts, yogurts and liqueurs. In addition, plants belonging to the genus *Ulex* are widely used in traditional Galician medicine, with *Ulex gallii* Planch. (Figure 1) being employed for the same anti-infective, hypotensive and diuretic purposes as *Ulex europaeus* L., which is the most studied species [1,2,3]. However, the majority of research on gorse has been conducted in relation to its varied applications in the treatment of cancer, which is considered one of the main public health problems in the world and is the second-leading cause of death worldwide. On one hand, lectins are a group of proteins that have the property of binding to different cell types such as tumor cells. For this reason, for decades they have served as a good tool for the diagnosis of different types of tumors. *U. europaeus* agglutinin (UEA-1) is known to be a specific lectin for some glycocompounds, and UEA-I binding can serve as a prognostic factor in ovarian cancer [4]. In the same way, it is used as a marker for the HER2 glycoprotein in breast cancer and as an endothelial marker in neoplasmic thyroid cells [5,6]. On the other hand, a water-soluble extract from *U. europaeus* seeds (European gorse) was found to inhibit the growth of various reticuloendothelial tumor cell lines via a noncytotoxic and reversible mechanism [7]. Furthermore, some types of myelomas were specifically sensitive to *U. europaeus* extracts [8]. Moreover, flower and leaf extracts of *Ulex parviflorus* L. have shown moderate cytotoxic activity in the A549 lung cancer cell line [9].

Antitumoral activities have been related to different compounds, such as formononetin, genistein and naringenin, identified in different *Ulex* species [10,11]. These compounds, which belong to the flavonoid family, interfere with multiple signal transduction pathways, resulting in the inhibition of proliferation, angiogenesis or metastasis, which are interesting properties in this field of study [12]. Flavonoids are secondary plant metabolites, responsible for the color of the flowers. They are also a part of the plant’s defense systems, given their anti-UV and antimicrobial properties.

Recently, the process of obtaining new active metabolites from plants has received great interest. However, isolating compounds from natural extracts is not always possible due to their chemical complexity and their presence in very low amounts. Metabolomics has emerged in recent years as an indispensable tool for the analysis of thousands of metabolites from crude natural extracts, leading to a paradigm shift in natural product drug discovery [13,14,15,16]. In particular, ultra-high-performance liquid chromatography (UHPLC), coupled with mass spectrometry analysis, has contributed substantially to improve the number of metabolites that can be detected, identified and quantified. Large datasets generated using these instruments are analyzed with the multivariate data analysis algorithms provided by bioinformatics tools. The information obtained is compared, also using bioinformatics tools, with several databases allowing researchers to assign metabolites and elucidate the chemical structures of novel compounds present in the extracts.

In this study, the chemical composition of the scarcely investigated polar extract from *U. gallii* was explored and its antitumor activity was evaluated towards the A549 (lung cancer) and AGS (stomach cancer) cell lines. According to the World Health Organization (WHO), lung and stomach cancer are among the top five most common causes of cancer deaths, causing 1.79 million and 769,000 deaths in 2020, respectively [17]. The survival rate for both is low and has not improved in recent times, indicating the urgent need to develop new therapeutic strategies. In this study we aimed to provide more scientific data for the application of *U. gallii* in fields such as the food and biomedicine industries.

## 2. Results

### 2.1. Extraction Yield

In the solid-liquid extraction process, using MeOH as solvent and starting with 369 g of the whole plant, 36.90 g of crude extract was obtained (10% yield). Liquid-phase extraction of the methanol crude extract afforded 2.60 g of the hexane fraction (H, 7.05%), 4.27 g of the dichloromethane fraction (D, 11.58%) and 26 g of the methanol fraction (M, 70.46%). H, D and M fractions were further fractionated, and as a result, we obtained twenty sub-fractions (Table 1).

### 2.2. Viability Assessment

**Figure 2:**
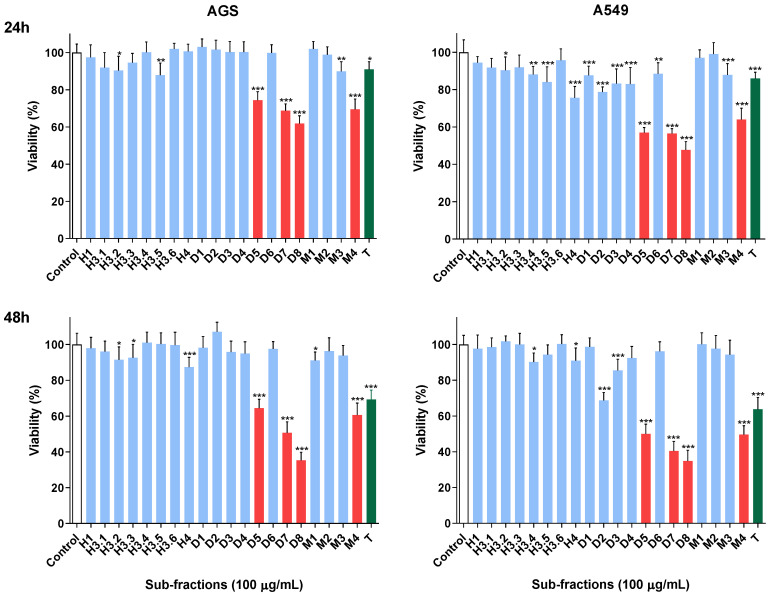
Effect of the sub-fractions (100 µg/mL) on the viability of AGS and A549 cells after 24 h (**top**) and 48 h (**bottom**) of exposure. Topotecan, T (0.5 µM), was used as a positive control for the analysis of cytotoxic effects. Data represent the means ± standard deviation (SD) of at least three independent experiments performed in triplicate. * *p* < 0.05, ** *p* < 0.01, *** *p* < 0.001.

AGS and A549 cell lines were initially treated for 24 h with the twenty sub-fractions of *U. gallii* obtained from the hexane (H), dichloromethane (D) and methanol (M) fractions at a concentration of 100 µg/mL. Sub-fractions D5, D7 and D8 (corresponding to the dichloromethane fraction) and M4 (corresponding to the methanol fraction) showed the greatest reduction in viability in both cell lines (Figure 2, top). At 48 h, we found overall that the sub-fractions that displayed more toxicity were the same as those identified at 24 h, although the effect was greater when the incubation time was higher (Figure 2, bottom). Based on these results, sub-fractions D5, D7, D8 and M4 were selected to be subjected to further mechanistic studies, namely, caspase activation and DNA quantification assessments in both cell lines, in order to try to elucidate the mechanisms involved in their toxicity.

### 2.3. Active Sub-Fractions Do Not Activate Effector Caspases but Reduce Proliferation

**Figure 3:**
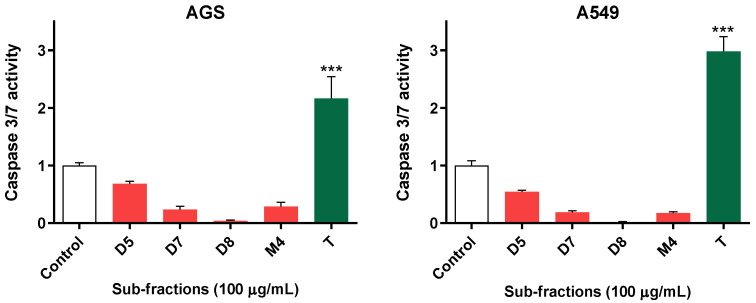
Effect of the sub-fractions (100 µg/mL) on the activity of caspase-3/7 of AGS and A549 cells after 24 h of exposure. Topotecan, T (0.5 µM) was used as a positive control. Data represent the means ± SD of at least three independent experiments performed in triplicate. *** *p* < 0.001.

Considering the significant effect of sub-fractions D5, D7, D8 and M4 on cell viability, we were interested in assessing whether some type of organized cell death could be taking place. To this end, we assessed the impact of the sub-fractions on the activity of executioner caspases, which are widely associated with many processes of organized cell death [18]. The results showed that, at the studied concentration (100 µg/mL), none of the tested sub-fractions increased caspase-3/7 activity in the studied cell lines. In both cases, topotecan was used as a positive control for caspase activation and a 2–3-fold increase in caspase activity was detected (Figure 3).

**Figure 4:**
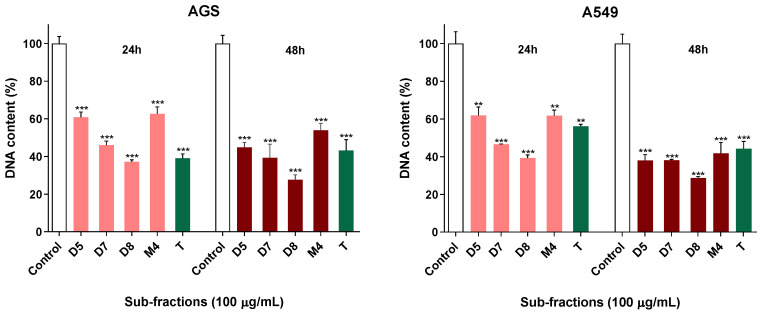
DNA content of AGS and A549 cells treated with sub-fractions (100 µg/mL) for 24 h and 48 h. Data represent the mean ± SD of at least three independent experiments performed in triplicate., ** *p* < 0.01, *** *p* < 0.001.

DNA quantification in AGS cells showed that treatment with sub-fractions D5, D7, D8 and M4 for 24 h reduced total DNA values by around 40%–50% compared to controls. This reduction was even greater when the treatment lasted for 48 h. Furthermore, a reduction in total DNA was also observed in A549 cells treated for 24 and 48 h with the same sub-fractions (Figure 4).

### 2.4. Identification of Metabolites via UHPLC-QToF Using Targeted/Untargeted Approaches

Considering the biological results, we proceeded with the characterization of bioactive compounds present in the active sub-fractions of *U. gallii*, whether they were flavonoids or potential new unidentified compounds. For this purpose, electrospray ionization (ESI) in negative mode was carried out and data acquisition was performed in a data-independent acquisition mode, detecting mainly pseudomolecular ions [M-H]^−^. Initially, a database with key parameters of specific compounds (flavonoids) present in *Ulex species* was constructed (Table S1) and the data were compared with those obtained experimentally for the different analytes. Therefore, firstly, a tentative identification was carried out in a targeted way, since the compounds present in the different sub-fractions were identified based on previous lists introduced in the software. The compounds tentatively identified, with the retention times and peak areas (%) for some of them, are presented in Table 2, Table 3, Table 4 and Table 5. The high sensitivity of the method used allowed us to tentatively identify metabolites that could not be associated with any peak in the chromatogram because of their low concentrations in the samples. Subsequently, an untargeted analysis, consisting of exhaustive analyses of the diverse compounds obtained, was performed using the Mass Bank of North America (Mona), Metaboscape and GNPS databases in order to tentatively identify a larger number of compounds than those identified in the targeted analysis.

**Figure 5:**
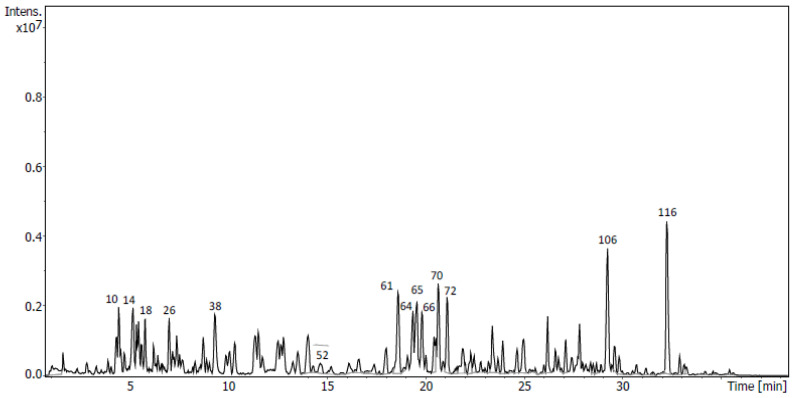
UHPLC-QToF chromatogram of sub-fraction D5 of *U. gallii*.

Targeted UHPLC-QToF analysis of the sub-fraction D5 allowed us to tentatively identify five compounds with a flavonoid structure (Figure 5, Table 2) based on the accurate masses of adduct [M-H]^−^ ions and their fragmentation patterns. These compounds have been previously reported as plant metabolites in *U. europaeus* [10]. The analysis indicated the presence of the flavanones dihydromyricetin and liquiritigenin, with their molecular ion peaks [M-H]^−^ at *m*/*z* 319.0461 and *m*/*z* 255.0665, respectively. It also indicated the presence of three isoflavones: isoprunetin, daidzein and genistein. Their [M-H]^−^ ions were identified at *m*/*z* 283.0613, *m*/*z* 253.0509, and *m*/*z* 269.0457 respectively. The fragment ion at *m*/*z* 151.0036 found for dihydromyricetin and genistein was inferred to be formed through the retro-Diels–Alder reaction, which is a basic fragmentation pathway of flavonoids (Figure S1) [19]. All the described polyphenols tentatively identified were found in very low percentages in the sub-fraction D5 and only dihydromyricetin and genistein could be correlated with peaks 26 and 52 of chromatogram, respectively.

**Figure 6:**
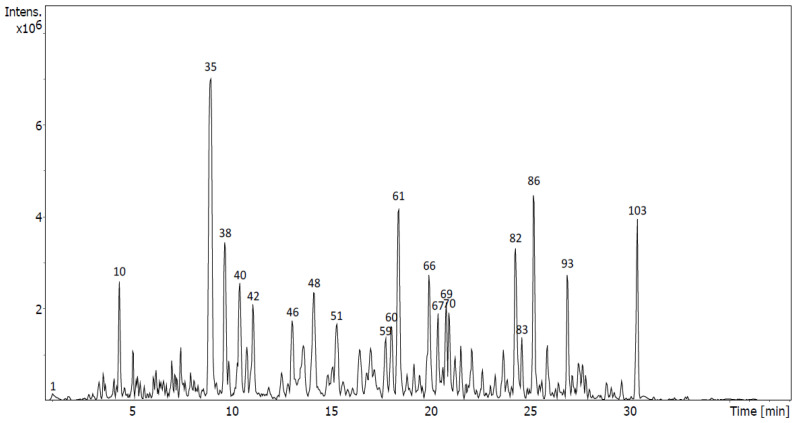
UHPLC-QToF chromatogram of sub-fraction D7 of *U. gallii*.

In sub-fraction D7, six plant metabolites with a flavonoid structure were tentatively identified (Figure 6, Table 3). Three of the metabolites corresponded to flavonoid structures linked to either two glucoses, such as quercetin-3,7-diglucoside with an [M-H]^−^ ion at *m*/*z* 625.1409, or one glucose: isoprunetin-7-*O*-glucoside and liquiritin, which displayed the [M-H]^−^ ions at *m*/*z* 445.1142 and *m*/*z* 417.1191, respectively. [M-H]^−^ ions also indicated the presence of three isoflavones—isoprunetin (peak 35) and daidzein (peak 38)—both of which were tentatively identified in D5. However, isoprunetin represented the highest peak in the chromatogram for D7, with an area of 11.72%. Additionally, formononetin (peak 61, area of 5.26%) was tentatively identified, with an [M-H]^−^ ion at *m*/*z* 267.0664 and a fragment ion at *m*/*z* 252.0429, formed by loss of a CH_3_ group, which is characteristic of its fragmentation pathway (Figure S2) [20].

**Figure 7:**
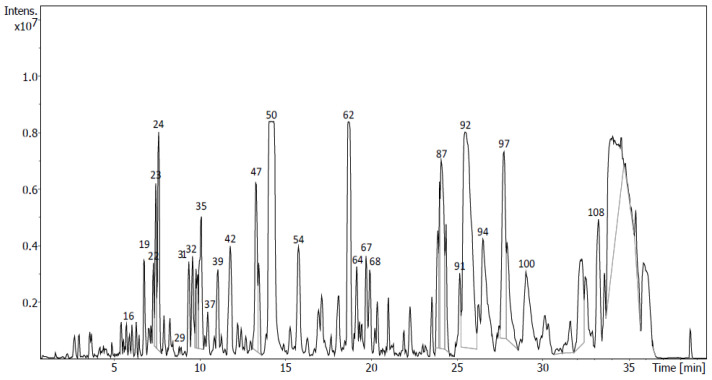
UHPLC-QToF chromatogram of sub-fraction D8 of *U. gallii*.

The targeted analysis of sub-fraction D8 allowed us to tentatively identify eleven compounds with a flavonoid structure (Figure 7, Table 4). Six of these compounds (isoprunetin-7-*O*-glucoside, formononetin, isoprunetin, liquiritin, genistein and liquiritigenin) were also tentatively identified in sub-fractions D5 and/or D7. However, it is noteworthy that the [M-H]^−^ ion peaks tentatively identified as genistein and liquiritigenin were higher in this sub-fraction. Genistein (peak 50, [M-H]^−^ at *m*/*z* 269.0459 and dimer [M-H]^−^ at *m*/*z* 539.0988) represented 9.38% of the total area of the chromatogram in this sub-fraction. In addition, it also occurred in its glycosylated form in this sub-fraction, forming genistin, which displayed an [M-H]^−^ ion at *m*/*z* 431.0988. The ion peak [M-H]^−^ assigned to liquiritigenin (peak 62) represented 4.60% of the total area. This metabolite displayed a precursor ion [M-H]^−^ at *m*/*z* 255.0666 and fragment ions [C_7_H_4_O_3_-H]^−^ at *m*/*z* 135.0088 and [C_8_H_8_O-H]^−^ at *m*/*z* 119.0503 that were likely produced via retro-Diels–Alder fragmentation in the C-ring of liquiritigenin [20]. The opening of its glycosylation product liquiritin (peak 31), with [M-H]^−^ at *m*/*z* 417.1193, was the origin of the chalcone neoisoliquiritin (peak 16) with [M-H]^−^ at *m*/*z* 417.1193. Both showed a fragment ion corresponding to aglycon liquiritigenin at *m*/*z* 255.0664. Furthermore, one flavonol linked to glucose, luteolin-4-*O*-glucoside, was tentatively identified with an [M-H]^−^ ion at *m*/*z* 447.0938. Apigenin was tentatively identified in this sub-fraction in its free (peak 37) or heterosidic form as apigenin-4-*O*-glucoside, with [M-H]^−^ at *m*/*z* 269.0458 and *m*/*z* 431.0988, respectively (Figure S3).

**Figure 8:**
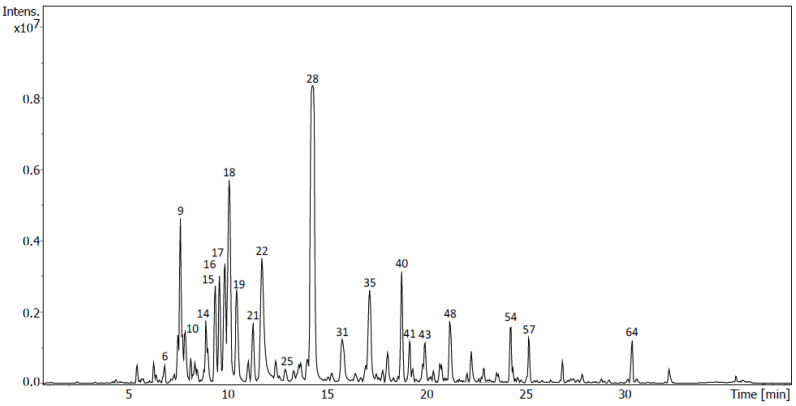
UHPLC-QToF chromatogram of sub-fraction M4 of *U. gallii*.

Finally, in sub-fraction M4, except for compounds tentatively identified as formononetin-*O*-glucoside, with [M-H]^−^ at *m*/*z* 429.1186, and naringenin (peak 25), with a precursor ion [M-H]^−^ at *m*/*z* 271.0613 and an ion fragment at *m*/*z* 151.0033, previously described as a naringenin fragment produced via the retro-Diels–Alder reaction, all the compounds tentatively identified here (Figure 8, Table 5) were also tentatively identified in sub-fractions D5, D7 and/or D8. As described for the sub-fraction D8, genistein (peak 28, 21.05%) and liquiritigenin (peak 40, 3.97%) are also the most abundant compounds tentatively identified in sub-fraction M4. Apigenin 4-*O*-glucoside (peak 6), with [M-H]^−^ at *m*/*z* 431.0986, showed an ion fragment at *m*/*z* 269.0457 corresponding to the aglycon apigenin. This isomer was assigned because its fragmentation pattern did not show the ion peak at *m*/*z* 413.0872 that is characteristic of apigenin-7-*O*-glucoside [21]. On the other hand, luteolin-*O*-glucoside, with [M-H]^−^ at *m*/*z* 447.0938, showed fragment ions at *m*/*z* 285.0407 and 286.0440, which have been associated with a luteolin-*O*-glucoside isomer, luteolin-4-*O*-glucoside (Figure S4) [22].

Untargeted analysis was performed with exhaustive analyses of all the diverse analytes present in the samples. Firstly, peak picking, deconvolution, peak alignment and compound annotation via peak matching against MS/MS public libraries was carried out using MS-Dial. Moreover, experimental fragmentation patterns were compared with those obtained in silico of compounds that were present in several databases of natural products using MS-Finder. Due to the complexity of the composition of the sub-fractions, only the highest peaks of the chromatograms (Figure 5, Figure 6, Figure 7 and Figure 8) were considered and submitted to MS-Finder. The suggested compounds, with their retention times, fragment ions and peak areas (%), are presented in Table 6. Through the above workflow, 57 metabolites were tentatively identified in the sub-fractions of *U. gallii* under study. Most of them correspond to flavonoids (40): 7 flavanones; 13 flavones, with one of them being a flavone glycoside; 6 flavonols; 13 isoflavones and 1 flavene.

Nine of 57 newly suggested metabolites had been previously tentatively identified in the targeted analysis. Additionally, untargeted analysis allowed the annotation of 10 prenylated flavonoids, most of them in the sub-fraction D7. Prenylation was identified in the following flavones: 6-prenylapigenin, with [M-H]^−^ at *m*/*z* 337.1084; licoflavone C, also with [M-H]^−^ at *m*/*z* 337.1084 (tentatively identified in regard to its fragmentation pattern), cyclomulberrin, with [M-H]^−^ at *m*/*z* 419.1504; and honyucitrin, with [M-H]^−^ at *m*/*z* 405.1710. It was also identified in the following isoflavones: 2,3-dehydrokievitol, 2,3-dehydrokievitone, gancaonin N and alpinumisoflavone with [M-H]^−^ at *m*/*z* 369.098, 353.1033, 367.1191 and 335.0929, respectively, and in the flavonols: licoflavonol, with [M-H]^−^ at *m*/*z* 353.1034, and 8-prenylkaempferol, also with [M-H]^−^ at *m*/*z* 353.1034, tentatively identified on the basis of characteristic fragmentation pattern analysis. In the most polar sub-fractions D8 and M4, kaempferol, with [M-H]^−^ at *m*/*z* 285.04054, and three methylated derivatives of kaempferol—rhamnocitrin, isokaempferide, with [M-H]^−^ at *m*/*z* 299.0562, and ermanin, with [M-H]^−^ at *m*/*z* 313.0719—were tentatively identified. Rhamnocitrin and isokaempferide were assigned in reference to their fragmentation pattern, with ion fragments at *m*/*z* 255.0664 and 256.0699 for isokaempferide, according to the literature [23]. In the sub-fraction M4, an ion peak [M-H]^−^ at *m*/*z* 285.0405 was tentatively associated with luteolin (peak 22). This is remarkable because this peak represented 7.79% of the total area of the chromatogram. In addition to flavonoids, other polyphenols such as coumarins, pterocarpanes, phenylbenzofuranes, perylenequinones and curcumins were also tentatively identified in different sub-fractions (Table 6).

Coumarins seemed to be more abundant in the D5 sub-fraction. 7-Ethoxy-4-methylcoumarin (peak 10; 2% of total area), mammeigin (peak 116; 6.62% of total area) and the coumarin glycoside 3,4,5-trihydroxy-6-[(4-methyl-2-oxo-2H-chromen-7-yl)oxy]oxane-2-carboxylic acid (peak 14; 2.55% of total area), with [M-H]^−^ at *m*/*z* 203.0714, 403.1552 and 351.0726, respectively, were tentatively identified in this sub-fraction. Additionally, in this sub-fraction three fatty acid derivatives were also tentatively identified: 11-hydroxy-9,10-dihydrojasmonic acid 11-beta-D-glucoside, 13-hydroxy-10-oxooctadec-11-enoic acid and 3-hydroxyhexadecanoic acid with [M-H]^−^ at *m*/*z* 389.1820, 311.2232 and 271.2280, respectively.

**Figure 9:**
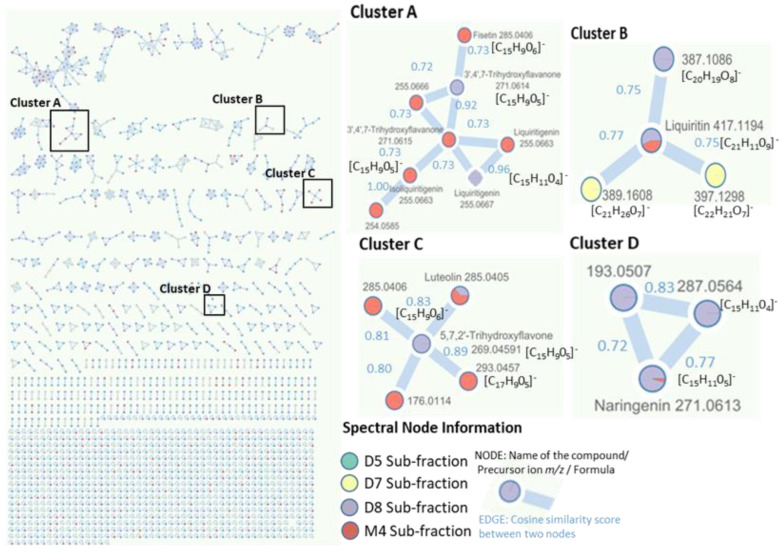
Molecular networking of the D5-8 and M4 sub-fractions as a complementary method to the targeted and untargeted analysis.

Molecular networking was employed to organize the metabolite candidates. This involved computing the spectral similarity between fragmentation spectra (using a cosine score) and visualizing the results as a network. Spectra were represented as “nodes”, and these nodes were connected by “edges”, forming a cluster when their similarities were above a similarity threshold. The UHPLC-QToF data from the four sub-fractions of *U. gallii* (D5, D7, D8 and M4) previously processed using MS-Dial were submitted for molecular networking through the GNPS web platform. The resulting molecular network comprised 2160 molecular features (nodes) and 272 independent clusters. Using spectral similarities, GNPS establishes relations between nodes, allowing one to group the metabolites with structural similarities (clusters) [24]. In cluster A (Figure 9), a liquiritigenin node ([C_15_H_11_O_4_]^−^ at *m*/*z* 255.0667) in the sub-fraction D8 was connected with another liquiritigenin node in the M4 sub-fraction and both were connected with a third node at *m*/*z* 271.0615 in the M4 sub-fraction, with a cosine similarity of 0.73. This means that the structure of the third node was quite similar to liquiritigenin, with the gain of an oxygen, and it was assigned to 3′,4′,7-trihydroxyflavanone via GNPS. At the same time, this third node was directly related to the other three nodes. One was identified as the same metabolite but in the D8 sub-fraction, and of the other two, with one less oxygen, one was assigned to isoliquiritigenin (*m*/*z* 255.0663), whereas the other could not be assigned. The 3′,4′,7-trihydroxyflavanone node in the sub-fraction D8 was directly related to a flavonoid in the sub-fraction M4 at *m*/*z* 285.0406 and was assigned to fisetin via GNPS (Cluster A, Figure 9).

In cluster B, liquiritin ([C_21_H_21_O_9_]^−^ at *m*/*z* 417.1194) was directly related to three other nodes. Two of these were present in the sub-fraction D7 at *m*/*z* 397.1298 and 389.1608 and the third in D8 at *m*/*z* 387.1086. Since GNPS did not assign a structure to these three nodes, the MS-Finder tool was applied to predict the molecular formulas associated with nodes [C_22_H_21_O_7_]^−^, [C_21_H_26_0_7_]^−^ and [C_20_H_19_O_8_]^−^, although the corresponding structures could not be assigned (Cluster B, Figure 9).

In cluster C, the node associated with 5,7,2′-trihydroxyflavone ([C_15_H_9_O_5_]^−^ at *m*/*z* 269.0458) in the sub-fraction D8 showed a cosine similarity score of 0.83 with a node at *m*/*z* 285.0405 in the sub-fraction M4. The difference of 16 mass units was probably due to the presence of one more oxygen [C_15_H_9_O_6_]^−^. The node was identified via GNPS as 3′,4′,5,7-tetrahydroxyflavone (luteolin), which coincided with the MS-Finder results. 5,7,2′-trihydroxyflavone was also related to three other more nodes in this cluster. One of these also had an *m*/*z* 285.0456, with a cosine value of 0.89, so this node could probably correspond to a positional isomer of 3′,4′,5,7-tetrahydroxyflavone. Another node had an *m*/*z* of 293.0457, with a difference of 24 mass units that could correspond to two carbons, so the corresponding ion could have the formula [C_17_H_9_O_5_]^−^. The third node, at *m*/*z* 176.0114, showed a large difference in mass, so it was not possible to establish a coherent formula based on the information obtained (Cluster C, Figure 9).

In cluster D, the naringenin in the sub-fractions D8 and M4 was assigned to a node at *m*/*z* 271.0613, related to two other nodes. One of them, with *m*/*z* 287.0564, could correspond to the formula [C_15_H_11_O_4_]^−^, even though the associated structure could not be predicted. The other node showed a greater difference in mass, so it was not possible to establish a coherent formula with the information obtained (Cluster D, Figure 9).

The flavonoids identified via targeted and untargeted analysis that did not appear in the highlighted clusters were nodes that did not have an established relationship with others (single nodes) or which belonged to other clusters for which we did not have information to tentatively identify further metabolites. Other matches were produced via GNPS, but these are not shown because they belonged to other families of compounds, such as fatty acids and sugars, that were in low proportions in the sub-fractions.

## 3. Discussion

In contrast to widely investigated *Ulex* species such as *U. europaeus*, there are no available records on the biological activity and chemical profile of *U. gallii*. Because antitumoral activity has been described for several *Ulex* species [7,8,9], the aim of this study was to help in discovering suitable and effective therapeutic sources against lung and stomach cancer. These are two of the most common types of cancer, with low survival rates. We performed a study of *U. gallii* metabolites guided by its in vitro activity on A549 (lung cancer) and AGS (stomach cancer) cell lines of various hexane (H1, H3.1–3.6 and H4), dichloromethane (D1–D8) and methanol (M1–M4) sub-fractions, which were obtained from a crude methanol extract. Three of the dichloromethane sub-fractions (D5, D7 and D8), as well as the methanol sub-fraction M4 (100 µg/mL), showed a time-dependent decrease in cell viability in both A549 and AGS cell lines. In addition, no cytotoxic activity was found for these sub-fractions in the MRC-5 cell line, indicating some selectivity (data not shown). The decrease in A549 and AGS cell viability caused by these sub-fractions was higher than that produced by topotecan (0.5 µM). Therefore, we were interested in assessing whether some type of organized cell death might be occurring.

Firstly, caspase-3/7 activity was evaluated in A549 and AGS cells treated with these sub-fractions. Caspase-3 and 7 executors are activated in the pathway of apoptosis. In this process, via the extrinsic pathway, after a stimulus, the death receptors are activated. They include Fas receptors, tumor necrosis factor receptors (TNF) and TNF-related apoptosis-inducing ligand receptors (TRAIL) that activate the cascade of caspases. Via the intrinsic pathway, after a stimulus, the mitochondrial outer membrane becomes permeabilized, resulting in apoptosome formation and the activation of caspase-9, which subsequently activates the caspase-3 executor [25]. In our experiments, none of the tested sub-fractions increased caspase-3/7 activity in the cell lines. Therefore, initially, it could be assumed that the toxicity generated by D5, D7, D8 and M4 subfractions does not trigger the pathway of apoptosis. Given the absence of an effect in terms of caspase activation, and considering the viability results, we hypothesized that the active fractions could be exerting their activity by the means of cytostatic effects, instead of cell death. Since cell proliferation also plays an essential role in carcinogenesis, which is considered a very important factor for cancer prevention [26], the search for effective agents that suppress or inhibit cell proliferation is the focus of many studies. Therefore, we evaluated the impact of the most active sub-fractions (D5, D7, D8 and M4) upon cell DNA contents, which is a good indicator of their proliferation status [27,28]. The total DNA values showed a time-dependent reduction of around 40–50% after treatment compared to controls in both A549 and AGS cell lines.

In order to explore the compounds that could potentially mediate the observed effects, our next aim was to tentatively identify metabolites present in the active sub-fractions through a targeted/untargeted analysis of UHPLC-ESI-QToF data. Previous phytochemical analyses of different *Ulex* species showed a high diversity of metabolite groups, such as fatty acids [29], carotenoids [30], flavonoids [31], pterocarpans [32] and alkaloids [33]. Because flavonoids are related to antitumoral activity, we addressed our study to investigating the flavonoid composition of *U. gallii*. A targeted analysis, using a database of 28 compounds (flavonoids) belonging to the *genus Ulex* obtained from literature (Table S1) and combining it with the DataAnalysis and TASQ software packages, as well as taking into account the exact mass and the deviation from the isotopic pattern as main criteria for validation, allowed us to tentatively identify a total of 17 compounds in the different sub-fractions (Table 2, Table 3, Table 4 and Table 5). Among them, dyhidromyricetin in the sub-fraction D5; isoprunetin, daidzein and formononetin in the sub-fraction D7; genistein and liquiritigenin in the sub-fraction D8; and apigenin, liquiritigenin and genistein in the sub-fraction M4were the most abundant. Untargeted analysis was performed using different metabolomic tools (MS-Dial, MS-Finder and GNPS), which provided information that could be compared to clarify the composition of the sub-fractions. Data files obtained via UHPLC-ESI-QToF were submitted to MS-DIAL software for data processing. MS-DIAL achieves compound identification through analyses of retention time, mass accuracy and isotope ratios, along with MS/MS similarity matching to libraries from publicly available databases [34]. The data obtained were exported to MS-FINDER, a universal program for compound annotation. This allowed us to tentatively identify a high number of flavonoids (Table 6), many of which have not been previously described in other species of the genus *Ulex.* They included flavanones such as dihydroisorhamnetin, dihydrokaempferol, isokaempferide and cryptostrobin; flavones such as oroxylin A, quercetin 3,3′-dimethyl ether, ermanin, dinatin, 5,7,2′-trihydroxyflavone, 5,7-dihydroxy-3′,4′,5′-trimethoxyflavone, 6-prenylapigenin, licoflavone C, cyclomulberrin and honyucitrin; isoflavones such as 5,7-dihydroxy-3-(6-hydroxy-1,3-benzodioxol-5-yl)chromen-4-one, pseudobabtigenin, 2,3-dehydrokievitol, neotenone, 6-hydroxy-7,4′-dimethoxyisoflavone, 2′,7-dihydroxy-4′,5′-dimethoxyflavone and 2,3-dehydrokievitone; and flavonols such as rhamnocitrin, rhamnazin, licoflavonol and 8-prenylkaempferol. Most of these were present in low percentages in the composition of *U. gallii* and only dihydrokaempferol in the sub-fractions D8 and M4, cyclomulberrin in the sub-fraction D5 and honyucitrin in the sub-fractions D7 and M4 exceeded 5% of the entire respective sub-fraction composition. The mentioned flavonoids might have contributed to the antiproliferative effects showed by the different sub-fractions [12].

Among the sub-fractions studied, the sub-fraction D5 showed the lowest composition of flavonoids. However, some of the flavonoids in this sub-fraction have been previously described for their antitumoral activity. The flavone cyclomulberrin, the most abundant flavonoid in this sub-fraction (peak 106), showed cytotoxic activity against Hep 3B (liver), MCF-7 (breast) and HeLA (cervical) cell lines [35]. Furthermore, 5,7-dihydroxy-3′,4′,5′-trimethoxyflavone, tentatively identified as peak 66 in this sub-fraction, previously showed a moderate cytotoxicity against the human lung carcinoma NCI-H460 cell line [36,37]. Nevertheless, no cytotoxic activity for these two flavonoids has been described in A549 and AGS cells. Dihydromyricetin, also tentatively identified in the sub-fraction D5 (peak 26), induces mitochondrial apoptosis in the A549 cell line via the suppression of reactive oxygen species-mediated ERK and JNK activation [38]. Researchers have also reported that it downregulated pro-caspase-3 in CNE-2 cells [39].

In the sub-fraction D-7, isoprunetin (peak 35) was tentatively identified as the most abundant flavonoid (11.72%). However, no antitumoral activity has been described for this compound. In this sub-fraction, we also tentatively identified for the first time in the *genus Ulex* several prenylated flavonoids that could explain the antitumoral activity found. It has been reported that flavonoids with prenyl groups are more cytotoxic than flavonoids without this structural feature [40]. 2,3-Dehydrokievitone (peak 66), which has a prenyl group at the C-8 position of the A-ring of the isoflavone, has been reported to exhibit strong cytotoxic activity and induce apoptosis efficiently in cancer cells [41]. Furthermore, the antitumoral activity of licoflavone C (peak 86) has been reported in human nasopharyngeal cancer cells via caspase activation, a mechanism of action that does not seem to be relevant to the antitumor activity of the sub-fraction D7 [42]. On the other hand, formononetin (peak 61), an isoflavone previously described in other *Ulex* species, elicits antitumorigenic properties in vitro and in vivo by modulating numerous signaling pathways. In agreement with our results, it has been reported that formononetin suppresses cell proliferation via signal transducer and activator of transcription (STAT) activation, as well as via phosphatidylinositol 3-kinase/protein kinase-B (PI3K/AKT) and the mitogen-activated protein kinase (MAPK) signaling pathway. It also induces cell apoptosis via an intrinsic pathway involving Bax, Bcl-2 and caspase-3 proteins and cell cycle arrest by regulating mediators such as cyclin A, cyclin B1 and cyclin D1, and inhibits cell invasion by regulating the growth factors vascular endothelial growth factor (VEGF) and fibroblast growth factor 2 (FGF2), as well as the proteins matrix metalloproteinase (MMP)-2 and MMP-9 [43].

In the sub-fraction D8, the isoflavone genistein (peak 50) was identified as the main flavonoid. It has been previously isolated from the roots of gorse (*U. europaeus*) and it is well known as an anticancer agent. It inhibits tumor cell proliferation and triggers cell cycle arrest and apoptosis in some cell types [44]. In adenocarcinoma (SK-BR-3) and ductal carcinoma (ZR-75-1) cells, it significantly inhibited proliferation in a dose-dependent manner by suppressing the expression of ERα and c-erbB-2 receptors [45]. Regarding A549 cells, it has been reported that genistein inhibits proliferation via miR-27a and MET signaling [46]. Genistein also showed antitumoral activity against AGS cells via the inhibition of growth related to the arrest of the cell cycle in the G2/M phase and via the downregulation of Cdc25C, which is a marker of cell proliferation [47]. On the other hand, the flavonone liquiritegin was the second most abundant flavonoid in this sub-fraction. The literature reports that high concentrations decrease the viability of both breast cancer MDA-MB-231 and BT549 cell lines by increasing apoptosis and enhancing caspase-3 activity in these cells [48]. However, this mechanism was not observed in our study when A549 and AGS cells were treated with the sub-fraction D8. Therefore, its antiproliferative activity must be due to other metabolites in this sub-fraction. Naringenin, also tentatively identified in this sub-fraction, has shown an inhibitory effect on human AGS cancer cells by activating autophagic proteins Beclin 1 and LC3B, with significant phosphorylation of mitogen-activated protein kinases (MAPKs) [49]. In the A549 cell line, it activates apoptosis by inducing caspase-3 cleavage, by arresting the cell cycle at G0/G1 and by suppressing cyclins A and D1 [50]. These and other antitumor effects described for other minor flavonoids in this sub-fraction, such as kaempferol, liquiritin, quercetin and 2,3-dehydrokievitone, could justify the greater antitumoral activity found for the sub-fraction D8 in our studies [41,51,52]. Genistein is also the most important flavonoid in the sub-fraction M4 (peak 28). In this sub-fraction, we also tentatively identified liquiritigenin (peak 40). The antitumoral effects of both have been described above. Additionally, dihydrokaempferol (peak 9) was tentatively identified in this sub-fraction. It has been described to show an antiproliferative effect on synovial cells promoting Bax and Bad expression and inhibiting Bcl-2 and Bcl-xL expression and initiating the caspase cascade [53]. In human malignant melanoma cells (SK-Mel-28), its antitumoral activity is mediated via the inhibition of cell migration and invasion and the upregulation of NF-kB/MAPK signaling pathways [54]. Isokaempferide (peak 17) and kaempferol (peak 18) have antimitotic effects (block cell growth by stopping mitosis, cell division) in HL-60 and CEM (leukemia), MCF-7 (breast), HCT-8 (colon) and B-16 (skin) tumoral cell lines. These two flavonoids differ only by the presence of a methoxyl group on C-3 of the C ring (isokaempferide) instead of a hydroxyl (kaempferol). Many authors have suggested that the presence of methoxyl substituents increases the cytotoxicity of flavonoids [55]. Luteolin (peak 22) has been reported to inhibit the growth of gastric cancer cells (AGS), leading to detention at the G2/M phase by reducing the protein levels of Cdc2, Cyclin B1 and Cdc25 [56].

## 4. Materials and Methods

### 4.1. Chemicals and Reagents

Dimethyl sulfoxide (DMSO), trypan blue, phalloidin–tetramethylrhodamine B isothiocyanate and topotecan hydrochloride were acquired from Sigma-Aldrich (St. Louis, MO, USA). Chloroform, dichloromethane, hexane and methanol were purchased from Merck (Darmstadt, Germany). Dulbecco’s modified Eagle’s medium (DMEM), Qubit™ 1X dsDNA assay Kit, PrestoBlue^TM^, Hank’s balanced salt solution (HBSS), fetal bovine serum (FBS), penicillin-streptomycin solution (penicillin 10,000 units/mL and streptomycin 10,000 μg/mL) and trypsin-EDTA (0.25%) were obtained from GIBCO, Invitrogen™ (Grand Island, NY, USA). A Caspase-Glo^®^ 3/7 luminescent kit was obtained from Promega Corporation. Sephadex LH-20 (17-0090-01) was purchased from Pharmacia Fine Chemicals.

### 4.2. Plant Material Collection, Preparation of Extract and Fractionation

**Figure 10:**
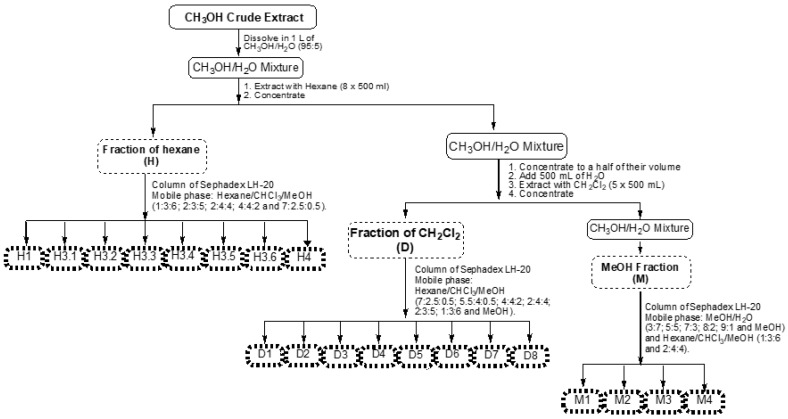
Process of obtaining the fractions and sub-fractions from *U. gallii*.

The whole plant was collected in Santa Mariña do Monte (San Sadurniño, Galicia) on 22 July 2018. It was identified as *U. gallii* Planch. and one of the samples was deposited in the Herbarium SANT USC, the Official Herbarium of the University of Santiago de Compostela, with the registration code SANT 76257. The entire plant was dried and powdered and then extracted with methanol over 48 h under agitation (600 rpm) at room temperature. The procedure was repeated three times, followed by the combination of the three methanol extracts. The crude extract obtained was evaporated to dryness and then redissolved in a mixture of methanol/H_2_O (95:5), after which it was subjected to liquid phase extraction using hexane (8 × 500 mL) to obtain the fraction of hexane (H). The remaining methanol/H_2_O mixture was concentrated to half of its volume, followed by addition of 500 mL of water to increase its polarity for better phase separation in the next extraction. Then, this mixture was extracted with dichloromethane (5 × 500 mL), obtaining the fraction of dichloromethane (D). The methanol/H_2_O mixture was evaporated to dryness, obtaining the methanol fraction (M). Fractions H and D were subsequently fractionated via Sephadex LH-20 column chromatography, using mixtures of hexane/chloroform/methanol (1:3:6; 2:3:5; 2:4:4; 4:4:2 and 7:2.5:0.5 for H and 7:2.5:0.5; 5.5:4:0.5; 4:4:2; 2:4:4; 2:3:5 and 1:3:6 followed by methanol for D) as a mobile phase-. In the same way, M was subsequently fractionated using mixtures of methanol/H_2_O (3:7; 5:5; 7:3; 8:2; 9:1), methanol and mixtures of hexane/chloroform/methanol (1:3:6 and 2:4:4). Eight sub-fractions were obtained from the hexane-partitioned fraction (H1, 3.1–3.6 and 4), 8 sub-fractions from the dichloromethane partitioned fraction (D1–8) and 4 sub-fractions from the methanol fraction (M1–4) (Figure 10).

### 4.3. Cell Culture

Human lung adenocarcinoma cell line A549 and human gastric cancer cell line AGS were purchased from Sigma-Aldrich (St. Louis, MO, USA) and cultured in DMEM + GlutaMAX^TM^ medium supplemented with 1% penicillin/streptomycin and 10% FBS, and maintained at 37 °C in a humidified atmosphere with 5% CO_2_. After reaching 80–90% confluence, cells were washed with fresh medium, trypsinized and subcultured. For the experiments, A549 and AGS cells were seeded in 96-well plates at a density of 1 × 10^4^ cells/well and 1.5 × 10^4^ cells/well, respectively, and maintained in an incubator under conditions of saturated humidity with a partial pressure of 5% CO_2_ in the air, at 37 °C.

### 4.4. Determination of Cell Viability

Cells were treated with sub-fractions H, D and M (100 μg/mL) or topotecan hydrochloride hydrate (0.5 µM) and incubated for 24 and 48 h. After this period, PrestoBlue^TM^, a commercial solution of resazurin, was added (1:10) and the fluorescence was monitored at 560/590 nm (excitation/emission wavelength) after 30 min using a Synergy H1 microplate reader. The anticancer molecule topotecan hydrochloride hydrate was used as positive control.

### 4.5. Caspase-3/7 Activity

The activity of caspase-3/7 was evaluated using the Caspase-Glo^®^ 3/7 kit assay. Cells were incubated with the D5, D7, D8 and M4 sub-fractions (100 µg/mL) or topotecan hydrochloride hydrate (0.5 µM) (positive control) in 96-well white plates, at 37 °C, for 24 h. Caspase-Glo^®^ 3/7 substrate was added to the same volume of cell supernatant and incubated for 30 min at 22 °C. After that, the luminescent signal was measured in a Sinergy H1 microplate reader.

### 4.6. DNA Quantification

After the treatment of A549 and AGS cells with D5, D7, D8 and M4 sub-fractions (100 µg/mL) or topotecan hydrochloride hydrate (0.5 µM) for 24 and 48 h, the Qubit^®^ dsDNA HS assay kit was used for DNA quantification according to the manufacturer’s instructions. Topotecan hydrochloride hydrate was used as positive control. The fluorescence was read in a Qubit^®^ 4 Fluorometer.

### 4.7. Ultra-High-Performance Liquid Chromatography Quadrupole Time-of-Flight Mass Spectrometry Analysis (UHPLC-QToF)

Compositional analysis was performed on a UHPLC system (Elute system; Bruker Corporation, Billerica, MA, USA) coupled with a quadrupole time-of-flight (QToF) mass spectrometer (Impact II; Bruker Corporation, Massachusetts, USA). The chromatographic separation of compounds was conducted with a Kinetex C18 column (2.6 µm, 100 mm × 2.1 mm, Phenomenex, Torrance, CA, USA), kept at a constant temperature of 35 °C, with a flow rate of 0.25 mL/min. The mobile phase consisted of 0.1% formic acid in water (A) and acetonitrile (B). The gradient method, expressed as a B percentage, was started at 5% for 5 min, and continued linearly to 20% for 10 min, then to 25% for 10 min, then to 50% for 20 min and finally to 100% for 5 min. It was returned to the initial conditions until reaching 55.20 min. The injection volume was set to 5 µL. Eluted components were ionized via electrospray ionization (ESI) in negative mode using N2 for nebulization (pressure of 2.5 Bar) and drying gas (flow of 6 L/min), a temperature of 200 °C and a capillary voltage of 4000 V. Data acquisition was performed using the broadband collision-induced dissociation (bbCID) method, a data-independent acquisition (DIA) method that provides MS and MS/MS spectra at the same time. All ions were fragmented using an alternating low collision energy (5 eV) to acquire MS spectra and a high collision energy (a voltage ramp from 10 to 30 eV) to acquire MS/MS spectra. The mass range was set at 50 to 1250 *m*/*z*. All acquisitions were obtained using Compass HyStar software (version 5.1) and initially processed and interpreted using the DataAnalysis and TASQ software packages (Bruker Corporation, Massachusetts, USA). All samples were studied combining the suspect screening approach (with suspected substances based on prior information) and the non-targeted screening approach.

#### 4.7.1. Targeted Screening

To tentatively identify some compounds in the samples, the algorithm of TASQ software was applied to a user-built database. Data from compounds present in species belonging to the genus *Ulex* (molecular formulas and exact masses of precursors and qualifier ions) were collected from the scientific literature and the main online databases of chemical compounds (PubChem, Mass Bank of North America (MoNA) and the Human Metabolome Database) and included in the user database (Table S1). The identification criteria were as follows: maximum acceptable deviation for exact masses (Δ*m*/*z*) of precursors and qualifier ions: 0.5 mDa, and isotopic pattern fit (mSigma) between the measured and the theoretical value: 50 mSigma.

#### 4.7.2. Untargeted Screening

Further data processing and metabolite annotation was carried out with freely available software supporting all ion fragmentation (AIF) technology. Bruker’s raw data files were converted to the ABF format using the Reifycs Abf (Analysis Base File) converter (https://www.reifycs.com/AbfConverter/ accessed on 30 November 2021). ABF files were submitted to metabolomics workflows using MS-DIAL software (http://prime.psc.riken.jp/compms/msdial/main.html; version 5.0.3, accessed on 10 December 2021) for data processing: peak picking, deconvolution, peak alignment and peak matching against MS/MS public libraries (mainly Mona, MassBank-EU, ReSpect, MetaboBASE and Global Natural Products Social Molecular Networking (GNPS)). The parameters were determined as follows: MS1 and MS2 tolerances: 0.02 and 0.05 Da, respectively; minimum peak height: 10,000; mass slice width: 0.05; sigma window value for deconvolution: 0.04; retention time and MS1 tolerances for peak alignment: 0.1 min and 0.02 Da, respectively; similarity peak matching score for compound annotation: higher than 70%. Data processed via MS-DIAL software were also converted into MSP and a MGF files in order to export the list of masses of interest from MS-DIAL to MS-Finder (http://prime.psc.riken.jp/compms/msfinder/main.html, accessed on 10 December 2021) and GNPS (https://gnps.ucsd.edu/ProteoSAFe/static/gnps-splash.jsp, accessed on 28 December 2021), respectively. MS-Finder software facilitates the annotation of unknown masses of interest by comparing their experimental fragmentation patterns with those obtained in silico by MS-Finder of compounds that are present in several databases of natural products (a screening approach which is useful when no appropriate experimental reference mass spectra are available for peak matching). The parameter settings were established as follows: MS1 and MS2 mass tolerance: 5 ppm and 15 ppm, respectively; potential candidates consisted only of C, H, O and N; and LipidMAPS, DrugBank, FooDB, PlantCyc, ChEBI, T3DB, STOFF, BLEXP, NPA, NANPDB and COCONUT were used as the selected databases. GNPS web software enabled further peak matching against several different spectral libraries and a feature-based molecular network (FBMN) analysis. To create networks of similar chemical structures, the parameter settings was established as follows: a mass tolerance of 0.05 Da was used for the parent mass, 0.05 Da for fragment ions, a cosine score above 0.7 for edges and more than 1 matched peaks. The nodes of networks were labeled with all the metabolite annotation information previously obtained with TASQ, MS-Dial, MS-Finder and GNPS and the entire molecular network was visualized using Cytoscape software (http://www.cytoscape.org, accessed on 28 October 2022).

### 4.8. Statistical Analysis

Data analysis was performed using GraphPad Prism 8.0 Software (San Diego, CA, USA). A one-way analysis of variance (ANOVA) was performed to detect significant differences between the variables, with a significance level of *p* < 0.05. All data were expressed as means ± standard deviation (SD).

## 5. Conclusions

In this study, several dichloromethane and methanol sub-fractions from *U. gallii* Planch showed antiproliferative activity against AGS and A549 cell lines. Targeted analysis of UHPLC-QToF data allowed us to tentatively identify a total of 17 flavonoid derivatives in these sub-fractions, with isoprunetin, genistein, formononetin and liquiritigenin being the most abundant. Through untargeted analysis, we tentatively identified 31 more flavonoids, including several prenylated flavonoids and kaempferol derivatives which have been described for the first time in the genus *Ulex* and some of them could contribute to the activity found.

The molecular networking process served to assist in the identification of metabolites in the plant extracts. Overall, the results showed that *U. gallii* can be considered a promising source of bioactive compounds with several therapeutic uses.

## Figures and Tables

**Table 1:** Percentage yields of sub-fractions of *U. gallii* with respect to the crude methanol extract.

Sub-Fractions	Sub-Fraction Weight (g)	Yield (%)
H1	0.70	1.90
H3.1	0.243	0.66
H3.2	0.110	0.30
H3.3	0.316	0.85
H3.4	0.118	0.32
H3.5	0.048	0.13
H3.6	0.292	0.79
H4	0.185	0.50
D1	0.232	0.63
D2	0.177	0.48
D3	0.141	0.38
D4	0.473	1.28
D5	0.229	0.62
D6	0.477	1.29
D7	1.333	3.61
D8	0.310	0.84
M1	6.296	17.06
M2	6.777	18.36
M3	6.796	18.42
M4	3.812	10.33

**Table 2:** Flavonoids identified in the targeted analysis by means of UHPLC-ESI-QToF of the sub-fraction D5 of *U. gallii* (Rt = retention time, *m*/*z* = mass/charge ratio of the compound, mSigma = deviation in the isotopic pattern, Δ*m*/*z* = deviation in the mass ratio/charge measured in relation to the expected value).

Flavonoids in D5	Rt (min)	Formula	*m*/*z* Measured	*m*/*z* Expected	Ion	mSigma	Δ*m*/*z* (mDa)	Peak Area (%)	Peak
Dihydromyricetin	7.00	C_15_H_12_O_8_	319.0461	319.0459	C_15_H_11_O_8_^−^	3.7	0.05	1.56	26
Isoprunetin	8.95	C_16_H_12_O_5_	283.0613	283.0612	C_16_H_11_O_5_^−^	2.8	0.06	-	-
Daidzein	9.70	C_15_H_10_O_4_	253.0509	253.0506	C_15_H_9_O_4_^−^	5.6	0.29	-	-
Genistein	14.30	C_15_H_10_O_5_	269.0457	269.0455	C_15_H_9_O_5_^−^	1.9	0.09	0.27	52
Liquiritigenin	18.75	C_15_H_12_O_4_	255.0665	255.0663	C_15_H_11_O_4_^−^	12	0.20	-	-

**Table 3:** Flavonoids identified in targeted analysis via UHPLC-ESI-QToF of sub-fraction D7 of *U. gallii* (Rt = retention time, *m*/*z* = mass/charge ratio of the compound, mSigma = deviation in the isotopic pattern, Δ*m*/*z* = deviation in the mass ratio/charge measured in relation to the expected value).

Flavonoids in D7	Rt (min)	Formula	*m*/*z* Measured	*m*/*z* Expected	Ion	mSigma	Δ*m*/*z* (mDa)	Peak Area (%)	Peak
Isoprunetin-7-*O*-glucoside	5.43	C_22_H_22_O_10_	445.1142	445.1140	C_22_H_21_O_10_^−^	6.6	0.18	-	-
Quercetin-3,7-diglucoside	6.52	C_27_H_30_O_17_	625.1409	625.1410	C_27_H_29_O_17_^−^	7.7	−0.15	-	-
Isoprunetin	8.95	C_16_H_12_O_5_	283.0613	283.0612	C_16_H_11_O_5_^−^	5.9	0.2	11.72	35
Liquiritin	9.33	C_21_H_22_O_9_	417.1191	417.1191	C_21_H_21_O_9_^−^	28.5	0.26	-	-
Daidzein	9.70	C_15_H_10_O_4_	253.0507	253.0507	C_15_H_9_O_4_^−^	7.2	0.17	3.97	38
Formononetin	18.40	C_16_H_12_O_4_	267.0664	267.0663	C_16_H_11_O_4_^−^	3.4	0.17	5.26	61

**Table 4:** Flavonoids identified in targeted analysis via UHPLC-ESI-QToF of sub-fraction D8 of *U. gallii* (Rt = retention time, *m*/*z* = mass/charge ratio of the compound, mSigma = deviation in the isotopic pattern, Δ*m*/*z* = deviation in the mass ratio/charge measured in relation to the expected value).

Flavonoids in D8	Rt (min)	Formula	*m*/*z* Measured	*m*/*z* Expected	Ion	mSigma	Δ*m*/*z* (mDa)	Peak Area (%)	Peak
Genistin	4.70	C_21_H_20_O_10_	431.0988	431.0984	C_21_H_19_O_10_^−^	14.4	0.48	-	-
Isoprunetin-7-*O*-glucoside	5.46	C_22_H_22_O_10_	445.1146	445.1140	C_22_H_21_O_10_^−^	6.6	0.62	-	-
Neoisoliquiritin	6.10	C_21_H_22_O_9_	417.1193	417.1191	C_21_H_21_O_9_^−^	1.7	0.33	0.21	16
Apigenin-4-*O*-glucoside	6.87	C_21_H_20_O_10_	431.0988	431.0984	C_21_H_19_O_10_^−^	13.0	0.41	-	-
Luteolin-4-*O*-glucoside	7.89	C_21_H_20_O_11_	447.0938	447.0933	C_21_H_19_O_11_^−^	4.6	0.52	-	-
Isoprunetin	8.94	C_16_H_12_O_5_	283.0614	283.0612	C_16_H_11_O_5_^−^	2.5	0.48	0.04	29
Liquiritin	9.40	C_21_H_22_O_9_	417.1193	417.1191	C_21_H_21_O_9_^−^	4.9	0.51	0.89	31
Apigenin	10.50	C_15_H_10_O_5_	269.0458	269.0455	C_15_H_9_O_5_^−^	4.4	0.45	0.42	37
Genistein	14.20	C_15_H_10_O_5_	269.0459	269.0455	C_15_H_9_O_5_^−^	4.4	0.45	9.38	50
Formononetin	18.38	C_16_H_12_O_4_	267.0664	267.0663	C_16_H_11_O_4_^−^	18.4	0.14	-	-
Liquiritigenin	18.70	C_15_H_12_O_4_	255.0666	255.0663	C_15_H_11_O_4_^−^	17.9	0.31	4.60	62

**Table 5:** Flavonoids identified in targeted analysis via UHPLC-ESI-QToF of sub-fraction M4 of *U. gallii* (Rt = retention time, *m*/*z* = mass/charge ratio of the compound, mSigma = deviation in the isotopic pattern, Δ*m*/*z* = deviation in the mass ratio/charge measured in relation to the expected value).

Flavonoids in M4	Rt (min)	Formula	*m*/*z* Measured	*m*/*z* Expected	Ion	mSigma	Δ*m*/*z* (mDa)	Peak Area (%)	Peak
Genistin	4.71	C_21_H_20_O_10_	431.0982	431.0984	C_21_H_19_O_10_^−^	7.5	−0.13	-	-
Isoprunetin-7-*O*-glucoside	5.46	C_22_H_22_O_10_	445.1139	445.1140	C_22_H_21_O_10_^−^	4.5	−0.16	-	-
Neoisoliquiritin	6.10	C_21_H_22_O_9_	417.1190	417.1191	C_21_H_21_O_9_^−^	6.3	−0.08	-	-
Apigenin-4-*O*-glucoside	6.80	C_21_H_20_O_10_	431.0986	431.0984	C_21_H_19_O_10_^−^	2.2	−0.13	0.55	6
Luteolin-4-*O*-glucoside	7.90	C_21_H_20_O_11_	447.0935	447.0933	C_21_H_19_O_11_^−^	2.8	−0.12	1.51	10
Formononetin-*O*-glucoside	8.82	C_22_H_22_O_9_	429.1186	429.1191	C_22_H_21_O_9_^−^	32.8	−0.55	-	-
Isoprunetin	8.90	C_16_H_12_O_5_	283.0612	283.0612	C_16_H_11_O_5_^−^	7.2	0.01	2.55	14
Liquiritin	9.40	C_21_H_22_O_9_	417.1193	417.1191	C_21_H_21_O_9_^−^	6.8	−0.01	2.84	15
Apigenin	10.50	C_15_H_10_O_5_	269.0457	269.0455	C_15_H_9_O_5_^−^	7.0	0.03	3.50	19
Naringenin	13.30	C_15_H_12_O_5_	271.0613	271.0612	C_15_H_11_O_5_^−^	7.3	0.05	0.26	25
Genistein	14.28	C_15_H_10_O_5_	269.0457	269.0455	C_15_H_9_O_5_^−^	15.0	0.12	21.05	28
Limonianin	18.42	C_20_H_16_O_5_	381.0097	381.0980	C_20_H_15_O_5_^−^	9.8	−0.04	-	-
Liquiritigenin	18.77	C_15_H_12_O_4_	255.0664	255.0663	C_15_H_11_O_4_^−^	10.5	0.08	3.97	40

**Table 6:** Metabolites identified via untargeted analysis in the different sub-fractions using MS-Dial and MS-Finder (Rt = retention time and *m*/*z* = mass/charge ratio of the metabolite).

Tentative Identification	Rt (min)	Formula	Ion	*m*/*z*	Fragment Ions (*m*/*z*)	Family	Peak Area (%)	Sub- Fraction	Peak
4-Hydroxybenzaldehyde	4.40	C_7_H_6_O_2_	C_7_H_5_O_2_^−^	121.0296	122.0329	Hydroxybenzaldehydes	2.28	D7	10
7-Ethoxy-4-methylcoumarin	4.50	C_12_H_12_O_3_	C_12_H_11_O_3_^−^	203.0714	204.0748,188.0479, 159.0454	Coumarins	2.00	D5	10
3,4,5-Trihydroxy-6-[(4-methyl-2-oxo-2H-chromen-7-yl)oxy]oxane-2-carboxylic acid	5.20	C_16_H_16_O_9_	C_16_H_15_O_9_^−^	351.0726	352.0759, 307.0826, 293.0670, 276.0597, 275.0563, 249.0770, 231.0664, 205.0872, 136.0168	Coumarin glycosides	2.55	D5	14
Dihydroisorhamnetin	5.80	C_16_H_14_O_7_	C_16_H_13_O_7_^−^	317.0669	318.0703, 293.0669, 289.0721, 245.0820, 230.0585, 205.0871, 190.0636	Flavanones	1.53	D5	18
3,4,9-Trihydroxypterocarpan	6.8	C_15_H_12_O_5_	C_15_H_11_O_5_^−^	271.0613	272.0647, 243.0663	Pterocarpans	0.86	D8	19
Dihydromyricetin	7.00	C_15_H_12_O_8_	C_15_H_11_O_8_^−^	319.0461	320.0495, 275.0562, 231.0663, 216.0428, 151.0036	Flavanones	1.56	D5	26
Unknown	7.30	C_16_H_12_O_6_	C_16_H_11_O_6_^−^	299.0562	300.0596	-	0.65	D8	22
Rhamnocitrin	7.50	C_16_H_12_O_6_	C_16_H_11_O_6_^−^	299.0562	599.1199, 300.0597	Flavonols	1.32	D8	23
Dihydrokaempferol	7.60	C_15_H_12_O_6_	C_15_H_11_O_6_^−^	287.0563	575.1198, 288.0597, 259.0613,125.0244	Flavanones	2.31/4.94	D8/M4	24/9
Isoprunetin	9.00	C_16_H_12_O_5_	C_16_H_11_O_5_^−^	283.0613	284.0647, 271.0613	Isoflavones	11.72/2.55	D7/M4	35/14
2-(3,4,5-Trihydroxyphenyl)-4H-chromene-3,4,5,7-tetrol	9.30	C_15_H_12_O_8_	C_15_H_11_O_8_^−^	319.0461	320.0495, 194.0176, 193.0142	Flavenes	2.86	D5	38
Liquiritin	9.40	C_21_H_12_O_9_	C_21_H_11_O_9_^−^	417.1193	835.2460, 418.1227, 329.0669, 255.0663	Flavone glycoside	0.89/2.84	D8/M4	31/15
Unknown	9.60	C_15_H_10_O_6_	C_15_H_9_O_6_^−^	285.0405	571.0885, 286.0440	-	0.95	D8	32
Unknown	9.60	C_15_H_10_O_6_	C_15_H_9_O_5_^−^	285.0406	571.0884, 286.0441	-	3.01	M4	16
Daidzein	9.70	C_15_H_10_O_4_	C_15_H_9_O_4_^−^	253.0507	507.1088, 254.0541	Isoflavones	3.97	D7	38
Isokaempferide	9.90	C_16_H_12_O_6_	C_16_H_11_O_6_^−^	299.0563	599.1195, 300.0597, 255.0664	Flavanones	3.59	M4	17
Kaempferol	10.10	C_15_H_10_O_6_	C_15_H_9_O_6_^−^	285.0405	571.0883, 286.0439	Flavonols	2.06/8.52	D8/M4	35/18
Oroxylin A	10.40	C_16_H_12_O_5_	C_16_H_11_O_5_^−^	283.0614	284.0648	Flavones	3.28	D7	40
Apigenin	10.50	C_15_H_10_O_5_	C_15_H_9_O_5_^−^	269.0457	539.0986, 270.0491	Flavones	3.50	M4	19
Maackiain	11.10	C_16_H_12_O_5_	C_16_H_11_O_5_^−^	283.0613	284.0648	Pterocarpans	2.44	D7	42
Quercetin 3,3′-dimethyl ether	11.10	C_17_H_14_O_7_	C_17_H_13_O_7_^−^	329.0669	659.1409, 330.0703	Flavones	0.91	D8	39
8-Hydroxy-7-(2-hydroxyphenyl)-9-methoxy-[1,3]dioxolo[4,5-g]chromen-6-one	11.3	C_17_H_12_O_7_	C_17_H_11_O_7_^−^	327.0512	655.1094, 328.0546, 283.0613	Coumarin	2.12	M4	21
Luteolin	11.70	C_15_H_10_O_6_	C_15_H_9_O_6_^−^	285.0405	571.0883, 286.0440	Flavones	7.79	M4	22
									
Ermanin	11.80	C_17_H_14_O_6_	C_17_H_13_O_6_^−^	313.0719	627.1509, 314.0754, 285.0406	Flavones	1.85	D8	42
Cryptostrobin	13.10	C_16_H_14_O_4_	C_16_H_13_O_4_^−^	269.0820	539.1711, 270.0854	Flavanones	2.34	D7	46
Naringenin	13.3	C_15_H_12_O_5_	C_15_H_11_O_5_^−^	271.0613	543.1296, 272.0648, 163.0038	Flavanones	2.41	D8	47
Rhamnazin	14.10	C_17_H_14_O_7_	C_17_H_13_O_7_^−^	329.0670	659.1409, 330.0703, 327.0513	Flavonols	3.86/0.91	D7/D8	48/39
Genistein	14.30	C_15_H_10_O_5_	C_15_H_9_O_5_^−^	269.0459	539.0988, 270.0491	Isoflavones	9.38/21.05	D8/M4	50/28
Dinatin	15.30	C_16_H_12_O_6_	C_16_H_11_O_6_^−^	299.0562	599.1197, 300.0597	Flavones	2.22	D7	51
3,3′,4′-Trihydroxyflavone	15.8	C_15_H_10_O_5_	C_15_H_9_O_5_^−^	269.0457	539.0985, 285.0406, 270.0490	Flavonol	2.57	M4	31
5,7,2′-Trihydroxyflavone	15.8	C_15_H_10_O_5_	C_15_H_9_O_5_^−^	269.0457	539.0989, 270.0492	Flavones	1.79	D8	54
5,7-Dihydroxy-3-(6-hydroxy-1,3-benzodioxol-5-yl)chromen-4-one	17.2	C_16_H_10_O_7_	C_16_H_9_O^7-^	313.0354	627.0781, 314.0389	Isoflavones	4.27	M4	35
Pseudobabtigenin	17.70	C_16_H_10_O_5_	C_16_H_9_O_5_^−^	281.0457	282.0492	Isoflavones	1.64	D7	59
2,3-Dehydrokievitol	18.00	C_20_H_18_O_7_	C_20_H_17_O_7_^−^	369.0983	739.2034, 370.1018	Isoflavones	2.02	D7	60
Formononetin	18.40	C_16_H_12_O_4_	C_16_H_11_O_4_^−^	267.0664	268.0698	Isoflavones	5.26	D7	61
11-Hydroxy-9,10-dihydrojasmonic acid 11-beta-D-glucoside	18.60	C_18_H_30_O_9_	C_18_H_29_O_8_^−^	389.1820	390.1855	Fatty acyl glycosides	3.89	D5	61
Liquiritigenin	18.80	C_15_H_12_O_4_	C_15_H_11_O_4_^−^	255.0666	511.1402, 256.0698, 135.0088	Flavanones	4.60/3.97	D8/M4	62/40
Neotenone	19.20	C_19_H_14_O_6_	C_19_H_13_O_6_^−^	337.0719	675.1506, 338.0753, 283.0613	Isoflavones	0.84 1.31	D8 M4	64 41
6-Hydroxy-7,4′-dimethoxyisoflavone	19.40	C_17_H_14_O_5_	C_17_H_13_O_5_^−^	297.0771	298.0805	Isoflavones	2.18	D5	64
2′,7-Dihydroxy-4′, 5′-dimethoxyflavone	19.60	C_17_H_14_O_6_	C_17_H_13_O_6_^−^	313.0720	314.0754, 299.0520, 298.0486	Isoflavone	3.24	D5	65
Justisolin	19.70	C_20_H_18_O_7_	C_20_H_17_O_7_^−^	369.0981	739.2033, 370.1015	Furanoid lignans	0.93	D8	67
5,7-Dihydroxy-3′,4′,5′-trimethoxyflavone	19.80	C_18_H_16_O_7_	C_18_H_16_O_6_^−^	343.0827	344.0860, 322.2029, 297.0408	Flavones	2.23	D5	66
2,3-Dehydrokievitone	19.90	C_20_H_18_O_6_	C_20_H_17_O_6_^−^	353.1033	707.2137, 354.1068, 351.0879, 297.0407	Isoflavones	3.66/0.9/1.7	D7/D8/ M4	66/68/43
Licoflavonol	20.40	C_20_H_18_O_6_	C_20_H_17_O_6_^−^	353.1034	707.2140, 354.1069	Flavonols	1.69	D7	67
UnKnown	20.60	C_19_H_34_O_5_	C_19_H_33_O_6_^−^	341.2337	683.4747, 342.2371	-	3.34	D5	70
Unknown	20.80	C_20_H_16_O_6_	C_20_H_15_O_6_^−^	351.0876	703.1824, 352.0910	-	1.66	D7	69
8-Prenylkaempferol	20.90	C_20_H_18_O_6_	C_20_H_17_O_6_^−^	353.1034	707.2141, 354.1069	Flavonols	1.52	D7	70
UnKnown	21.10	C_19_H_34_O_5_	C_19_H_33_O_6_^−^	341.2336	342.2370	-	2.82	D5	72
3,6a,7,10-Tetrahydroxy-4,9-dioxo-4,5,6,6a,6b,7,8,9-octahydroperylene	21.2	C_20_H_16_O_6_	C_20_H_15_O_6_^−^	351.0876	703.1823, 352.0910	Perylenequinones	2.50	M4	48
13-Hydroxy-10-oxooctadec-11-enoic acid	23.40	C_18_H_32_O_4_	C_18_H_31_O_4_^−^	311.2232	312.2266, 247.0977	Lineolic acids and derivatives	1.85	D5	81
N-(2-methylpropyl)-3-phenyl-2-(3-{2,5,9-trimethyl-7-oxo-3-phenyl-7H-furo[3,2-g]chromen-6-yl}propanamido)propanamide	24.1	C_36_H_38_N_2_O_5_	C_36_H_37_N_2_O_5_^−^	577.2698	578.2729, 279.1640	Phenylbenzofurans	3.88	D8	87
6-Prenylapigenin	24.2	C_20_H_18_O_5_	C_20_H_17_O_5_^−^	337.1084	675.2238, 338.1118	Flavones	1.75	M4	54
Demethoxycurcumin	24.30	C_20_H_18_O_5_	C_20_H_17_O_5_^−^	337.1086	675.2242, 338.1120	Curcuminoids	4.36	D7	82
Gancaonin N	24.60	C_21_H_20_O_6_	C_21_H_19_O_6_^−^	367.1191	735.2450, 368.1225	Isoflavones	1.06	D7	83
Licoflavone C	25.20	C_20_H_18_O_5_	C_20_H_17_O_5_^−^	337.1084	675.2241, 338.1118	Flavones	4.55	D7	86
p-Hydroxycinnamoyl-feruloylmethane	25.20	C_20_H_18_O_5_	C_20_H_17_O_5_^−^	377.1086	633.1410, 338.1121	Curcuminoids	1.41	M4	57
Unknown	25.20	C_31_H_40_N_4_O_7_	C_31_H_39_N_4_O_7_^−^	579.2850	580.2884, 476.2786, 339.1243	-	0.77	D8	91
Unknown	25.5	C_34_H_40_N_2_O_5_	C_34_H_39_N_2_O_5_^−^	555.2848	1111.5769, 556.2881, 353.2006	-	10.68	D8	92
Candidusin C	26.20	C_21_H_18_O_6_	C_21_H_17_O_6_^−^	365.1033	366.1067	Phenylbenzofurans	1.84	D5	92
Unknown	26.50	C_25_H_30_N_6_O_2_	C_25_H_29_N_6_O_2_^−^	445.2363	891.4801, 446.2398, 323.1292, 277.2176	-	3.63	D8	94
Alpinumisoflavone	26.90	C_20_H_16_O_5_	C_20_H_15_O_5_^−^	335.0929	671.1932, 336.0964	Isoflavone	2.59	D7	93
Unknown	27.70	C_25_H_32_N_6_O_2_	C_25_H_31_N_6_O_2_^−^	447.2520	895.5114, 448.2553, 279.2332	-	3.95	D8	97
3-Hydroxyhexadecanoic acid	27.80	C_16_H_32_O_3_	C_16_H_32_O_3_^−^	271.2280	272.2314	Long-chain fatty acids	1.83	D5	99
15-(4-Hydroxy-2,2,6-trimethyl-7-oxabicyclo[4.1.0]heptan-1-yl)-4,9,13-trimethyl-14-oxopentadeca-2,4,6,8,10,12-hexaenal	29.0	C_27_H_36_O_4_	C_27_H_35_O_4_^−^	423.2521	424.2555, 255.2332	Sesterterpenoids	2.96	D8	100
Cyclomulberrin	29.20	C_25_H_24_O_6_	C_25_H_23_O_6_^−^	419.1504	420.1538, 388.1488, 387.1453	Flavones	5.04	D5	106
Honyucitrin	30.40	C_25_H_26_O_5_	C_25_H_25_O_5_^−^	405.1710	406.1744	Flavones	3.95/1.23	D7/M4	103/64
Mammeigin	32.20	C_25_H_24_O_5_	C_25_H_23_O_5_^−^	403.1552	404.1586, 807.3179	Coumarins	6.62	D5	116
UnKnown	33.20	C_51_H_58_N_12_	C_51_H_58_N_12_^−^	837.4838	838.4871	-	2.45	D8	108

## Data Availability

Not applicable.

## Supplementary Materials

The following supporting information can be downloaded at: https://www.mdpi.com/article/10.3390/molecules28010351/s1, Figure S1: Mass spectrum corresponding to dihydromyricetin (top, peak 26, Rt: 7.00 min) and genistein (bottom, peak 52, Rt: 14.30) in the sub-fraction D5; Figure S2: Mass spectrum corresponding to formononetin (peak 61, Rt: 18.40 min) in the sub-fraction D7; Figure S3: Mass spectrum corresponding to apigenin (top, peak 37, Rt: 10.50 min) and apigenin-4-*O*-glucoside (bottom, Rt: 6.87) in the sub-fraction D8; Figure S4: Mass spectrum corresponding to luteolin-4-*O*-glucoside (peak 10, Rt: 7.90 min) in the sub-fraction M4. Table S1: Database of flavonoids present in *Ulex* species.

## Sample Availability

Sub-fractions of *U. gallii* are available from the authors.
