# Supplementary material for: Phytochemical Analysis and Antiproliferative Activity of Ulex gallii Planch. (Fabaceae), a Medicinal Plant from Galicia (Spain)
Source: Molecules. 2023 Jan 1;28(1):351. doi: 10.3390/molecules28010351 (PMC9822445; doi:10.3390/molecules28010351)
Supplement: Supplementary file 1 [file molecules-28-00351-s001.zip › molecules-2092922-supplementary.pdf]

Supplementary Material

Phytochemical Analysis and Antiproliferative Activity of *Ulex gallii* Planch. (Fabaceae), a Medicinal Plant from Galicia (Spain)

Lucía Bada [1,2], Renato B. Pereira [3], David M. Pereira [3,*], Marta Lores [4], María Celeiro [5], Elías Quezada [6], Eugenio Uriarte [6,7], José Gil-Longo [2] and Dolores Viña [1,2,*]

1. Group of Pharmacology of Chronic Diseases (CD Pharma), Molecular Medicine and Chronic Diseases Research Centre (CIMUS), Universidade de Santiago de Compostela, 15782-Santiago de Compostela, Spain
2. Department of Pharmacology, Pharmacy and Pharmaceutical Technology, Faculty of Pharmacy, Universidade de Santiago de Compostela, 15782-Santiago de Compostela, Spain
3. REQUIMTE/LAQV, Laboratório de Farmacognosia, Departamento de Química, Faculdade de Farmácia, Universidade do Porto, 4050-313 Porto, Portugal
4. Laboratory of Research and Development of Analytical Solutions (LIDSA), Department of Analytical Chemistry, Nutrition and Food Science, Faculty of Chemistry, Universidade de Santiago de Compostela, 15782-Santiago de Compostela, Spain
5. CRETUS Institute, Department of Analytical Chemistry, Nutrition and Food Science, Campus Vida, Universidade de Santiago de Compostela, 15782-Santiago de Compostela, Spain
6. Department of Organic Chemistry, Faculty of Pharmacy, Universidade de Santiago de Compostela, 15782-Santiago de Compostela, Spain
7. Instituto de Ciencias Químicas Aplicadas, Universidad Autónoma de Chile, 7500912 Santiago, Chile

* Correspondence: dpereira@ff.up.pt (D.M.P.); mdolores.vina@usc.es (D.V.); Tel.: +35-122-042-8655 (D.M.P.); +34-881-815-424 (D.V.)

Table S1. Database of flavonoids present in *Ulex* species.

Metabolite	Formula	Type	Mode	Ion	Collision Energy	*m/z*	Relative intensity	Database	Reference
Isowighteone	$C_{20}H_{18}O_5$	LC-ESI-QTOF	[M-H]$^-$	$C_{20}H_{17}O_5^-$	10 eV 20 eV	177.0188, 319.0970, 337.1076/ 177.0188, 295.0970, 307.0970, 319.0970, 337.1076	2.7847, 3.68867, 86.9560/ 44.8500, 2.2009, 4.2065, 3.2897, 48.8165	HMDB (Predicted)	https://hmdb.ca/spectra/ms_ms/158859 https://hmdb.ca/spectra/ms_ms/158860
Limonianin	$C_{20}H_{16}O_5$	LC-ESI-QTOF	[M-H]$^-$	$C_{20}H_{15}O_5^-$	10 eV 20 eV	217.0501, 309.0763, 317.0814, 335.0919/ 191.0708, 251.0344, 319.0606, 335.0919	0.6327, 0.8304, 0.5542, 94.8013/ 2.0200, 5.5959, 2.2507, 74.3874	HMDB (Predicted)	https://hmdb.ca/spectra/ms_ms/155106 https://hmdb.ca/spectra/ms_ms/155107
Isoprunetin	$C_{16}H_{12}O_5$	LC-ESI-QTOF	[M-H]$^-$	$C_{16}H_{11}O_5^-$	20V	196.0486, 240.0427, 268.0371, 283.0608	7.2022, 92.7647, 95.2782, 100.0000	MoNA (Experimental)	https://mona.fiehnlab.ucdavis.edu/spectra/display/VF-NPL-QTOF004611
Dihydromyricetin	$C_{15}H_{12}O_8$	LC-ESI-QTOF	[M-H]$^-$	$C_{15}H_{11}O_8^-$	10 eV 20 eV	151.0032, 153.0188, 301.0348, 319.0454/ 125.0239, 151.0031, 301.0348, 319.0454	7.2372, 3.1921, 3.0270, 67.0856/ 7.6694, 15.6284, 4.6647, 25.8881	HMDB (Predicted)	https://hmdb.ca/spectra/ms_ms/22583 https://hmdb.ca/spectra/ms_ms/22584
Genistein	$C_{15}H_{10}O_5$	LC-ESI-QTOF LC-ESI-ITTOF	[M-H]$^-$	$C_{15}H_9O_5^-$	10 eV 20 eV	133.0274, 181.0631, 201.0554, 269.04355 269.0400, 269.3396, 270.0498, 271.0455, 540.1046	5.6, 2.7, 1.5, 100/ 1.906, 5.2535, 3.7033, 4.5508, 19.4843	MoNA (Experimental) HMDB (Experimental)	https://mona.fiehnlab.ucdavis.edu/spectra/display/RIKENPlaSMA005451 https://hmdb.ca/spectra/ms_ms/6394
Quercetin 3,7-diglucoside	$C_{27}H_{30}O_{17}$	LC-ESI-QTOF	[M-H]$^-$	$C_{27}H_{29}O_{17}^-$	30 eV	301.0000, 463.000, 505.0000	20.00, 100.00,10.00	MoNA (Experimental)	https://mona.fiehnlab.ucdavis.edu/spectra/display/PM016302
Isoprunetin-7-O-glucoside	$C_{22}H_{22}O_{10}$	LC-ESI-QTOF	[M-H]$^-$	$C_{22}H_{21}O_{10}^-$	-	282.0514, 283.0586, 445.1175	100.0000, 82.9099, 4.0310	MoNA (Experimental)	https://mona.fiehnlab.ucdavis.edu/spectra/display/VF-NPL-QTOF005060
Neoisoloquiritin	$C_{21}H_{22}O_9$	LC-ESI-QTOF	[M-H]$^-$	$C_{21}H_{21}O_9^-$	10 eV 20 eV	119.0497, 237.0552, 255.0657, 417.1186 119.0497, 237.0552, 255.0657, 417.1186	3.8957, 4.1872, 21.2357, 31.6361 5.8093, 6.4842, 37.4759, 4.5595	HMDB (Predicted)	https://hmdb.ca/spectra/ms_ms/137952 https://hmdb.ca/spectra/ms_ms/137953
Daidzein	$C_{15}H_{10}O_4$	UHPLC-ESI-QTOF	[M-H]$^-$	$C_{15}H_9O_4^-$	30 eV	223.0453, 224.0506, 253.0501, 253.3113	12.21, 11.81, 100.00, 8.71	HMDB (Experimental)	https://hmdb.ca/spectra/ms_ms/5886
Inermin	$C_{16}H_{12}O_5$	LC-ESI-QTOF	[M-H]$^-$	$C_{16}H_{11}O_5^-$	10 eV 20 eV	237.0552, 253.0501, 265.0501,283.0606 237.0551, 253.0500, 265.0500, 283.0606	2.3492, 2.7754, 1.2463, 88.7910 7.7075, 13.6947, 6.7467, 54.4447	HMDB (Predicted)	https://hmdb.ca/spectra/ms_ms/22295 https://hmdb.ca/spectra/ms_ms/22296

Formononetin	$C_{16}H_{12}O_4$	LC-ESI-QTOF	[M-H]$^-$	$C_{16}H_{11}O_4^-$	10 eV	223.409, 251.0328, 252.0426, 267.0662	1.4404, 30.8938, 100.0000	MoNA (Experimental)	https://mona.fiehnlab.ucdavis.edu/spectra/display/VF-NPL-QTOF007716
Genistin	$C_{21}H_{20}O_{10}$	LC-ESI-QTOF	[M-H]$^-$	$C_{21}H_{19}O_{10}^-$	10 eV<br>20 eV	103.0395, 269.0450, 413.0872, 431.0978<br>73.0290, 269.0450, 413.0873, 431.0978	3.1718, 28.8188, 3.7217, 38.9856<br>3.1659, 7.4359, 3.5405, 5.7824	HMDB (Predicted)	https://hmdb.ca/spectra/ms_ms/19073<br>https://hmdb.ca/spectra/ms_ms/19074
Soyasapogenol B	$C_{30}H_{50}O_3$	LC-ESI-QTOF	[M-H]$^-$	$C_{30}H_{49}O_3^-$	10 eV<br>20 eV	421.3470, 427.3576, 439.3576, 457.3682<br>427.3576, 439.3576, 441.3368, 457.3681	4.2356, 5.0888, 23.4589, 51.2316<br>4.0092, 27.7025, 1.1133, 21.81467	HMDB (Predicted)	https://hmdb.ca/spectra/ms_ms/164916<br>https://hmdb.ca/spectra/ms_ms/164917
Resokaempferol	$C_{15}H_{10}O_5$	LC-ESI-QTOF	[M-H]$^-$	$C_{15}H_{15}O_5^-$	10 eV<br>20 eV	269.0449, 270.0476, 271.0469<br>224.0447, 239.0320, 269.0408, 270.436	100.00, 12.61, 1.70<br>1.70, 2.30, 100.00, 10.41	HMDB (Experimental)	https://hmdb.ca/spectra/ms_ms/373875<br>https://hmdb.ca/spectra/ms_ms/373876
Luteolin-4-O-glucoside	$C_{21}H_{20}O_{11}$	LC-ESI-QTOF	[M-H]$^-$	$C_{21}H_{19}O_{11}^-$	10 eV	285.0388, 286.0418, 287.0416, 441.0925	100.00, 17.80, 2.60, 68.20	MoNA (Predicted)	https://mona.fiehnlab.ucdavis.edu/spectra/display/RIKENPlaSMA005697
Luteolin-7-O-glucoside	$C_{21}H_{20}O_{11}$	LC-ESI-QTOF	[M+FA-H]$^-$	$C_{21}H_{19}O_{11}^-$	10 eV	284.0297, 284.0559, 285.0418, 447.0932	15.0847, 12.4764, 23.7698, 100.0000	MoNA (Experimental)	https://mona.fiehnlab.ucdavis.edu/spectra/display/CCMSLIB00000846983
Quercetin-O-glucoside	$C_{21}H_{20}O_{12}$	LC-ESI-QTOF	[M-H]$^-$	$C_{21}H_{19}O_{11}^-$	-	255.0274, 271.0261, 300.0258, 301.0351, 463.0889	10.39, 23.35, 100.00, 87.02, 98.06	HMDB (Experimental)	https://hmdb.ca/spectra/ms_ms/440558
Eriodictyol 7-O-glucoside	$C_{21}H_{22}O_{11}$	LC-ESI-QTOF	[M-H]$^-$	$C_{21}H_{21}O_{11}^-$	10 eV	287.0533, 288.0582, 289.0566, 449.1077	100.00, 15.40, 2.40, 23.30	MoNA (Experimental)	https://mona.fiehnlab.ucdavis.edu/spectra/display/RIKENPlaSMA006155
Rutin	$C_{27}H_{30}O_{16}$	LC-ESI-QTOF	[M-H]$^-$	$C_{27}H_{29}O_{16}^-$	10 eV<br>20 eV	163.0606, 301.0348, 591.1349, 609.1455<br>272.0281, 301.0344, 609.1456	6.3662, 18.8843, 6.0445, 20.6713<br>16.00, 91.00,99.00	HMDB (Predicted)<br>MassBank.EU (Experimental)	https://hmdb.ca/spectra/ms_ms/273297<br>https://massbank.eu/MassBank/RecordDisplay?id=MSBNK-Washington_State_Univ-BML00141&dsn=Washington_State_Univ
Liquiritin	$C_{21}H_{22}O_9$	LC-ESI-QTOF	[M-H]$^-$	$C_{21}H_{21}O_8^-$	10 eV<br>20 eV	237.0551, 255.0657, 399.1079, 417.1185<br>161.0449, 237.0551, 255.0865, 417.1186	4.5417, 24.9801, 3.3249, 34.4403<br>3.5209, 5.8702, 43.2606, 4.9646	HMDB (Experimental)	https://hmdb.ca/spectra/ms_ms/168057<br>https://hmdb.ca/spectra/ms_ms/168058
Apigenin 4-O-glucoside	$C_{21}H_{20}O_{10}$	LC-ESI-QTOF	[M+FA-H]-	$C_{21}H_{19}O_{10}^-$	20eV	268.0384, 269.0450, 431.0997	17.6810, 91.3639, 100.0000	MoNA (Experimental)	https://mona.fiehnlab.ucdavis.edu/spectra/display/CCMSLIB00000847310
Apigenin 7-O-glucoside	$C_{21}H_{20}O_{10}$	LC-ESI-QTOF	[M-H]$^-$	$C_{21}H_{19}O_{11}^-$	10 eV<br>20eV	103.0395, 269.0449, 413.0872, 431.0978	3.0945, 27.6401, 9.3458, 37.5765	HMDB (Predicted)	https://hmdb.ca/spectra/ms_ms/148638

						251.0344, 269.0449, 413.0872, 431.0978	3.2514, 55.1915, 6.6065, 5.8183		https://hmdb.ca/spectra/ms_ms/148639
Kaempferol-5-O-glucoside	$C_{21}H_{20}O_{11}$	LC-ESI-QTOF	$[M-H]^-$	$C_{21}H_{19}O_{11}^-$	10 eV<br>20eV	267.0293, 285.0399, 429.0821, 447.0927<br>161.0449, 267.0293, 285.0399, 429.0821	4.2709, 24.7430, 8.0195, 33.3460<br>3.5939, 6.0272, 45.6949, 5.1339	HMDB (Predicted)	https://hmdb.ca/spectra/ms_ms/158397<br>https://hmdb.ca/spectra/ms_ms/158398
Formononetin-O-glucoside	$C_{22}H_{22}O_9$	LC-ESI-QTOF	$[M-H]^-$	$C_{22}H_{21}O_9^-$	10 eV<br>20eV	103.0395, 267.0657, 411.1079, 429.1185<br>237.0551, 267.0657, 411.1079, 429.1185	3.1818, 28.4173, 7.3022, 38.9445<br>5.8294, 52.7782, 5.6049, 5.6364	HMDB (Predicted)	https://hmdb.ca/spectra/ms_ms/17537<br>https://hmdb.ca/spectra/ms_ms/17538
Kaempferol	$C_{15}H_{10}O_6$	UHPLC-ESI-QTOF	$[M-H]^-$	$C_{15}H_9O_5^-$	-	119.0343, 164.9985, 285.0399	79.4299, 38.6044, 33.5242	HMDB (Experimental)	https://hmdb.ca/spectra/ms_ms/6081
Naringenin	$C_{15}H_{12}O_5$	UHPLC-ESI-QTOF	$[M-H]^-$	$C_{15}H_{11}O_5^-$	-	93.0339, 119.0501, 151.0038, 271.0606	7.61, 60.76, 60.06, 100.00	HMDB (Experimental)	https://hmdb.ca/spectra/ms_ms/5790
Apigenin	$C_{15}H_{10}O_5$	LC-ESI-QTOF	$[M-H]^-$	$C_{15}H_9O_5^-$	10 eV<br>20eV	269.0458, 270.0491, 271.0507<br>151.0035, 227.0350, 269.0461	29.184, 4.562, 6.100<br>1.222, 3.906, 6.014	MassBank.EU (Experimental)	https://massbank.eu/MassBank/RecordDisplay?id=MSBNK-Fiocruz-FIO00011&dsn=Fiocruz<br>https://massbank.eu/MassBank/RecordDisplay?id=MSBNK-BGC_Munich-RP015112&dsn=BGC_Munich
Liquiritigenin	$C_{15}H_{12}O_4$	LC-ESI-QTOF	$[M-H]^-$	$C_{15}H_{11}O_4^-$	10 eV	119.0520, 135.0123, 255.0697, 255.2351	50.5431, 69.5440, 100.0000, 21.7868	MoNA (Experimental)	https://mona.fiehnlab.ucdavis.edu/spectra/display/MoNA018159

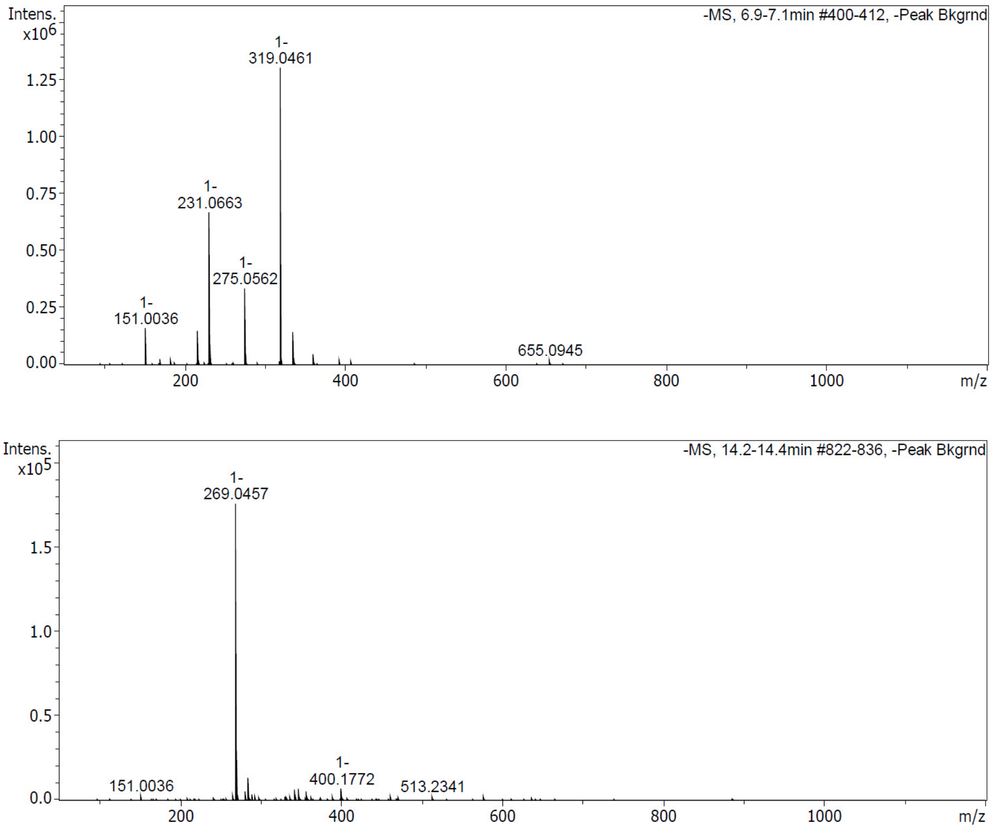

Figure S1. Mass spectrum corresponding to dihydromyricetin (top, peak 26, Rt: 7.00 min) and genistein (bottom, peak 52, Rt: 14.30) in D5 sub-fraction.

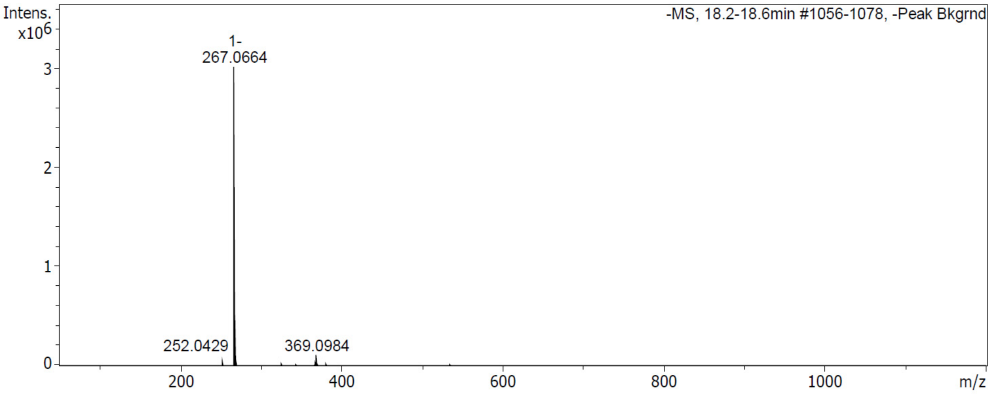

Figure S2. Mass spectrum corresponding to formononetin (peak 61, Rt: 18.40 min) in D7 sub-fraction.

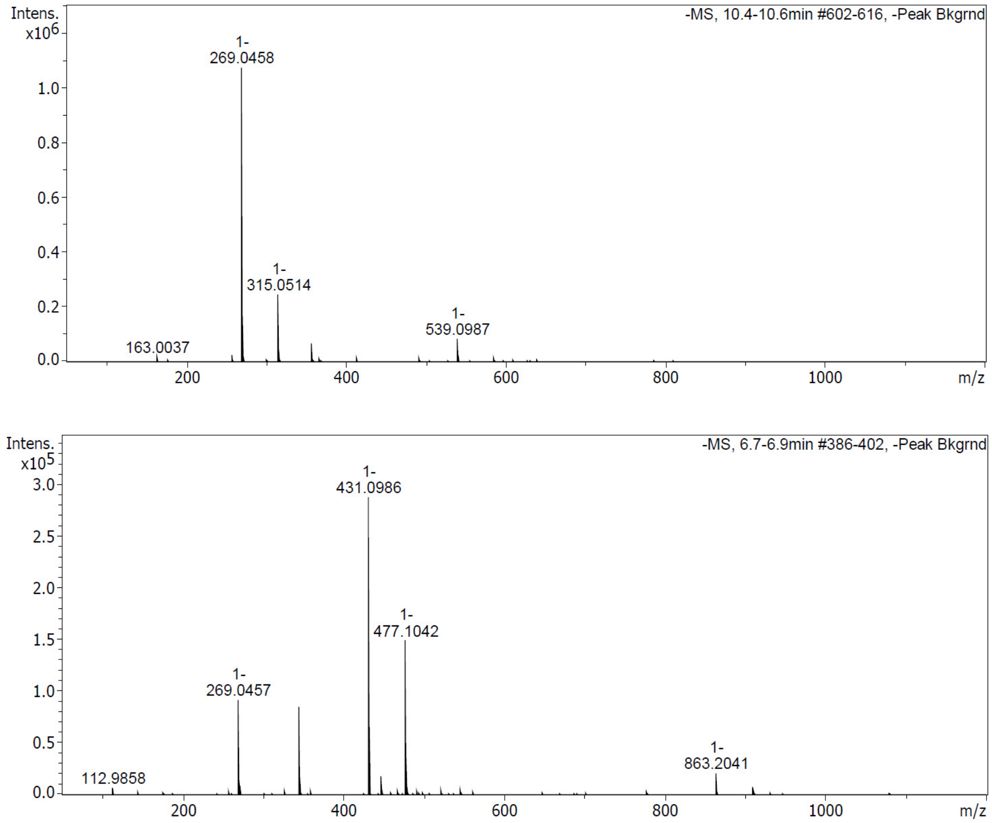

Figure S3. Mass spectrum corresponding to apigenin (top, peak 37, Rt: 10.50 min) and apigenin-4-*O*-glucoside (bottom, Rt: 6.87) in D8 sub-fraction.

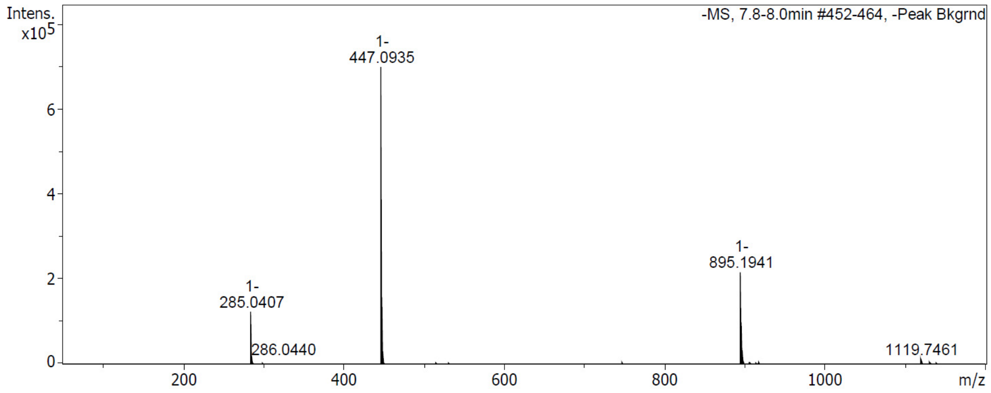

Figure S4. Mass spectrum corresponding to luteolin-4-*O*-glucoside (peak 10, Rt: 7.90 min) in M4 sub-fraction.
